# 1-Aminocyclopropane-1-Carboxylate Oxidase Induction in Tomato Flower Pedicel Phloem and Abscission Related Processes Are Differentially Sensitive to Ethylene

**DOI:** 10.3389/fpls.2017.00464

**Published:** 2017-03-31

**Authors:** Marko Chersicola, Aleš Kladnik, Magda Tušek Žnidarič, Tanja Mrak, Kristina Gruden, Marina Dermastia

**Affiliations:** ^1^Department of Biotechnology and Systems Biology, National Institute of BiologyLjubljana, Slovenia; ^2^Jožef Stefan International Postgraduate SchoolLjubljana, Slovenia; ^3^Department of Biology, Biotechnical Faculty, University of LjubljanaLjubljana, Slovenia; ^4^Department of Forest Physiology and Genetics, Slovenian Forestry InstituteLjubljana, Slovenia

**Keywords:** abscission, ACO, cell separation, ethylene, laser microdissection, programmed cell death, tomato, ultrastructure

## Abstract

Ethylene has impact on several physiological plant processes, including abscission, during which plants shed both their vegetative and reproductive organs. Cell separation and programmed cell death are involved in abscission, and these have also been correlated with ethylene action. However, the detailed spatiotemporal pattern of the molecular events during abscission remains unknown. We examined the expression of two tomato *ACO* genes, *LeACO1*, and *LeACO4* that encode the last enzyme in ethylene biosynthesis, 1-aminocyclopropane-1-carboxylate oxidase (ACO), together with the expression of other abscission-associated genes involved in cell separation and programmed cell death, during a period of 0–12 h after abscission induction in the tomato flower pedicel abscission zone and nearby tissues. In addition, we determined their localization in specific cell layers of the flower pedicel abscission zone and nearby tissues obtained by laser microdissection before and 8 h after abscission induction. The expression of both *ACO* genes was localized to the vascular tissues in the pedicel. While *LeACO4* was more uniformly expressed in all examined cell layers, the main expression site of *LeACO1* was in cell layers just outside the abscission zone in its proximal and distal part. We showed that after abscission induction, ACO1 protein was synthesized in phloem companion cells, in which it was localized mainly in the cytoplasm. Samples were additionally treated with 1-methylcyclopropene (1-MCP), a competitive inhibitor of ethylene actions, and analyzed 8 h after abscission induction. Cell-layer-specific changes in gene expression were observed together with the specific localization and ethylene sensitivity of the hallmarks of cell separation and programmed cell death. While treatment with 1-MCP prevented separation of cells through inhibition of the expression of polygalacturonases, which are the key enzymes involved in degradation of the middle lamella, this had less impact on the occurrence of different kinds of membrane vesicles and abscission-related programmed cell death. In the flower pedicel abscission zone, the physical progressions of cell separation and programmed cell death are perpendicular to each other and start in the vascular tissues.

## Introduction

The small volatile hydrocarbon ethylene is a gaseous plant hormone that affects diverse physiological processes during plant growth and development, including abscission (Ruduś et al., 2013). Abscission facilitates the shedding of organs that are no longer needed, infected, damaged, or senescent, and it promotes the falling of ripe fruit and the dispersal of seeds. Abscission occurs in a systemically regulated way at the predetermined abscission zone in response to developmental, hormonal, and environmental cues (Roberts et al., 2002; Leslie et al., 2007). In many studied systems, including tomato (Kimura and Sinha, 2008; Klee and Giovannoni, 2011; Ito and Nakano, 2015), the timing of abscission is determined by tissue sensitivity to ethylene, as ethylene can induce or accelerate abscission (Roberts et al., 2002). When ethylene is a signal for abscission, the rate at which abscission occurs depends upon the antagonistic effects of auxins and ethylene (Meir et al., 2010, 2015).

The last step in biosynthesis of ethylene is the conversion of 1-aminocyclopropane-1-carboxylic acid (ACC) to ethylene, which is catalyzed by the enzyme 1-aminocyclopropane-1-carboxylate oxidase (ACO) (Van de Poel et al., 2012; Ruduś et al., 2013). Expression of the *ACO* genes is tightly linked to the ethylene levels produced by the plant (Van de Poel et al., 2012; Ruduś et al., 2013). In tomato, there are seven *ACO* genes (Supplementary Figure 1; Seymour et al., 2013), with *LeACO1* and *LeACO4* showing the highest expression in tomato flower and fruit tissues (The Tomato Genome Consortium, 2012; Tomato eFP Browser^1^). A transcript of *LeACO1* is induced early after flower removal in the pedicel abscission zone (Meir et al., 2010; Kim et al., 2015), while *LeACO4* expression is high in the pedicel abscission zone before abscission induction, after which it drops off (Kim et al., 2015). *LeACO5* has been shown to be expressed in the tomato flower pedicel distal to the abscission zone area (Nakano et al., 2013), as well as in the abscission zone (Meir et al., 2010). However, expression of *LeACO1* and *LeACO4* in specific cell layers of the flower pedicel abscission zone and the localization of the ACO protein within the abscission zone are not known. Moreover, the subcellular localization of the ACO protein remains unclear (Reinhardt et al., 1994; Ramassamy et al., 1998; Chung et al., 2002).

In the tomato pedicel abscission zone, the involvement of ethylene in abscission has been demonstrated for the cell-separation process, which is characterized by major induction of cell-wall-modifying and hydrolytic enzymes. This results in degradation of the middle lamella between the cells in the abscission zone, which then allows the physical separation of the abscised organ from the mother plant (Meir et al., 2010; Tucker and Kim, 2015). In addition, spatially and temporally regulated ethylene signaling can induce programmed cell death (PCD) in sensitized cells or tissues of many plants (Trobacher, 2009).

We recently demonstrated that the activation phase of abscission in tomato leaf includes two spatially separated processes: cell layers proximal to the abscission zone are characterized by several features of membrane vesicular trafficking and by induction of the cell-wall hydrolyzing polygalacturonases, while hallmarks of PCD occur primarily distal to the abscission zone (Bar-Dror et al., 2011; Dermastia et al., 2012). We additionally showed that the involvement of PCD in abscission is through expression of the *LeLX* gene, which encodes a T2/S-like ribonuclease LX, an ortholog of the Arabidopsis developmental PCD marker gene *Ribonuclease 3* (*RNS3*) (Olvera-Carrillo et al., 2015). The transcript of *LeLX* and its encoded protein LX are preferentially localized at the distal side of abscission zone of the flower pedicel and leaf petiole (Bar-Dror et al., 2011). Moreover, leaf abscission is delayed in antisense tomato plants that have reduced levels of LX (Lers et al., 2006). The distal side of the tomato flower abscission zone is also characterized by specific expression of *Tomato bifunctional nuclease* (*LeTBN1*) gene and by the activity of a nuclease that appears to be encoded by this gene (Bar-Dror et al., 2011). *LeTBN1* is an ortholog of Arabidopsis *Bifunctional nuclease 1* (*BFN1*), which is another gene that is included in a developmental PCD cluster (Olvera-Carrillo et al., 2015). The requirement for PCD or abscission has also been supported by delayed abscission in tomato plants that overexpress the genes for the anti-apoptotic proteins Sf IAP and p35 (Bar-Dror et al., 2011). The induction of PCD usually includes increased levels of reactive oxygen species, which are mediators of stress signals (Petrov et al., 2015). As additional support for the association of PCD with abscission, following the induction of pedicel abscission, high levels of reactive oxygen species have been reported for the cells on the surface of the exposed abscission-zone fractures (Bar-Dror et al., 2011). Furthermore, an important regulatory role of reactive oxygen species in abscission has been recently shown for cassava leaf abscission under water-deficit stress (Liao et al., 2016).

In the present study we elucidated a spatiotemporal distribution of the key players involved in the tomato flower pedicel abscission in specific cell layers of the pedicel abscission zone or its close vicinity. We addressed the question of specifically where is the last enzyme in the ethylene biosynthetic pathway, ACO, localized, at both the transcriptional and protein levels and clearly showed it in the phloem tissues. We also examined the responsiveness of several genes involved in cell separation and PCD to ethylene during flower pedicel abscission. The results show that they were differentially affected by ethylene in a spatiotemporal manner, which was also confirmed at the ultrastructural level.

## Materials and methods

The experimental overview of this study is shown in Supplementary Figure 2.

### Plant growth conditions and treatments

Wild-type tomato plants (Solanum lycopersicum cv. VF36) were grown in growth chambers at 25°C and 75% relative humidity, and under a 16/8-h day/night cycle. A day before each experiment, all of the young flowers that had not reached full opening and the opened and senescent flowers were removed from the plants. For the experiments, only the flowers that had opened the night before the initiation of the experiments were used. All of the experiments were performed using detached flower clusters with up to four flowers, which were kept in 150 mL water in Erlenmeyer flasks. Abscission was induced by removal of the flowers from their pedicels with a sharp razor blade, as previously described (Meir et al., 2010). The samples were collected at predefined times: 0 h, as before flower removal, and 2, 4, 6, 8, 10, and 12 h after flower removal.

The treatment of the detached flower clusters with 1-methylcyclopropene (1-MCP) was performed by exposing the flower clusters to an atmosphere of 1 μL L^−1^ 1-MCP in air-tight 50-L chambers for 2 h prior to the induction of abscission. Control pedicels were sampled before abscission induction and remaining pedicels attached to the flower clusters were again exposed to 1-MCP for 8 h and then sampled. The 1-MCP was prepared from 0.14% SmartFresh powder (AgroFresh Inc., Rohm and Haas Company) mixed with water (80 mg in 2 mL), resulting in an estimated concentration of 1,000 ppb (v/v) 1-MCP in the treatment chamber, according to the manufacturer's instructions. This concentration completely inhibited pedicel abscission for the duration of the experiment.

### Histological sections

After induction of abscission, the pedicels were excised with a razor blade and fixed in 3.7% formaldehyde, 50% ethanol, 5% glacial acetic acid, overnight at 4°C. The samples were then dehydrated through an increasing series of ethanol and tertiary butyl alcohol concentrations, and embedded in Paraplast Plus (Sherwood Medical). Then 10-μm-thick longitudinal sections were cut on a rotary microtome (Autocut 2,040; Reichert-Jung), and placed on Superfrost Ultra Plus slides (Menzel-Gläser).

### Manual tissue dissection, laser microdissection, and gene expression analysis

Thin layers of tissue were excised for gene expression analysis of each side of the pedicel abscission-zone fracture using a scalpel, and frozen in liquid nitrogen (Supplementary Figure 2). These samples were homogenized in Tissuelyser (Qiagen). Total RNA was extracted using RNeasy Micro kits (Qiagen), according to manufacturer's protocol.

Laser microdissection was performed using a laser dissection microscope (PALM MicroBeam; Zeiss). The consecutive tissue sections of the same samples that were used for light microscopy and immunolocalization, were placed on MembraneSlide 1.0 PEN slides (Zeiss), deparaffinized in xylene, with the water removed from the tissue using 100% ethanol. Specific tissue regions of the pedicel (i.e., distal, distal abscission zone, proximal, proximal abscission zone, distal veins, proximal veins, distal pith, proximal pith), were dissected from consecutive tissue slices (*n* = 5) and collected in AdhesiveCap 500 plastic tubes (Zeiss). The tubes were snap frozen in liquid nitrogen and stored at −80°C. Total RNA was isolated from the tissue samples using RNeasy FFPE kits (Qiagen), according to the manufacturer's instructions.

Quantitative reverse transcription real-time PCR (qPCR) was performed on samples from three biological replicates in a PCR machine (ABI ViiA 7TM Real-Time PCR System; Applied Biosystems). The gene expression assays were designed using the Custom TaqMan Gene Expression Assays service (Applied Biosystems), as applied to: *LeACO1* (Solyc07g049530) and *LeACO4* (Solyc02g081190), which encode ACO; *LeEIL2* (Solyc01g009170), which encodes the ortholog of the *Arabidopsis* transcription factor *EIN3; LeRBOH1* (Solyc08g081690), which encodes NADPH oxidase; *LeTAPG1* (Solyc02g067630) and *LeTAPG4* (Solyc12g096750), which encode tomato abscission-related polygalacturonases 1 and 4, respectively; *LeLX* (Solyc05g007940), which encodes LX ribonuclease; and *LeTBN1* (Solyc02g078910), which encodes a nuclease (Supplementary Table 1). Expression assays (at 20 × concentrations) and the One-step RT-PCR AgPath ID mastermix reagents (Life Technologies) were used according to the manufacturer's instructions. The qPCR was performed in a final reaction volume of 5 μL, which contained 2 μL RNA and 3 μL reaction mix.

The relative expression of the target and reference genes were determined using the standard curve quantification method (Pfaffl, 2006; Žel et al., 2012). An RNA pool of abscission-zone samples was used to prepare the standard sample from which the standard calibration curve was measured. Every sample was tested in two dilutions for each gene, and the relative copy numbers were calculated from the calibration curve. All of the genes were normalized relative to the expression of the reference gene, *COX* (Bar-Dror et al., 2011; Müller et al., 2015), which encodes cytochrome oxidase, and the gene expression values are given as relative copy numbers. For visualization of the time series expression profiles, the time-point data were normalized to the control (time point, 0). Two-way ANOVA was used to evaluate the effects on gene expression of the factors “time after induction” and “abscission-zone side” and the interactions between these two factors (*p* < 0.05). The data were log2 transformed prior to the analysis. Statistical analysis was performed using the R software environment, version 3.1.2 (Supplementary Table 2). *Post-hoc* analysis of the results for gene expression data was performed using the Tukey's Honest Significant Difference method (Supplementary Tables 2.1–2.8).

### Immunolocalization

The sections were dewaxed in xylene and rehydrated in a decreasing ethanol series, and finally in TBST (1 × TBS pH 7.6, 0.2% Tween-20). Antigen retrieval was performed by incubating the slides in sodium citrate buffer (10 mM sodium citrate, pH 6.0) for 15 min in a boiling water bath. The sections were incubated in blocking solution (TBST plus 5% normal donkey serum) for 30 min at room temperature, followed by incubation with the 1:100 dilution of ACO1 primary antibody (Santa Cruz Biotechnology, aN-19) in blocking solution for 1 h at room temperature. The ACO1 polyclonal antibody used was raised in goat against a sequence between amino acids 1–50 of ACO of *Arabidopsis thaliana* (accession# Q06588), which shares 80% identity with ACO1 of *S. lycopersicum*. Non-immune goat serum was used for the negative controls (Supplementary Figure 3). After washing for 3 × 10 min in TBST, the sections were incubated with 1:1,000 alkaline-phosphatase-conjugated anti-goat antibodies (Jackson ImmunoResearch) in blocking solution for 1 h at room temperature, followed by washing as above. The staining was developed in NBT/BCIP substrate solution (Roche Diagnostics), and the sections were washed with water, dehydrated rapidly through an increasing ethanol series, followed by xylene, and mounted in Permount (Electron Microscopy Sciences). The stained sections were observed with a microscope (Zeiss AxioImager Z1) and photographed using a color digital camera (AxioCam HRc). Immunolocalization of Tomato abscission-related polygalacturonase (*TAPG4*) was performed essentially as described above, with affinity purified anti-TAPG4 polyclonal antibodies (BioGenes Antibodies) raised in rabbit against a peptide sequence 306–320 (CPNHESCPNQGSGVK) of TAPG4 (accession# Q96488). The blocking solution was TBST plus 5% normal goat serum, primary antibody was diluted 1:100 in blocking solution and the secondary antibody was 1:1,000 alkaline-phosphatase-conjugated anti-rabbit (Jackson ImmunoResearch) in blocking solution. Non-immune rabbit serum was used for the negative controls (Supplementary Figure 3). Washing and detection steps were performed as above.

### Detection of nuclear DNA fragmentation by TUNEL assay

Terminal deoxynucleotidyl transferase dUTP nick-end labeling (TUNEL) assays (Gavrieli et al., 1992) for detection of chromosomal DNA fragmentation were performed in the histological tissue sections using ApopTag Fluorescein *in-Situ* Apoptosis Detection kit (Millipore). The rehydrated sections were treated with 100 μg mL^−1^ proteinase K (Qiagen) for 15 min at room temperature, and incubated in a mixture of digoxigenin-labeled deoxynucleotides and terminal deoxynucleotidyl transferase for 60 min at 37°C. This was followed by incubation with fluorescein-labeled anti-digoxigenin antibodies. The slides were washed with phosphate-buffered saline and mounted using FluoroMount (Sigma-Aldrich), with 600 nM 4′-6-diamidino-2-phenylindole (DAPI). The fluorescein-labeled nuclei were observed under blue-light excitation (excitation, 450–490-nm band-pass; emission, 515-nm long-pass), and the DAPI fluorescence of all of the nuclei was observed under UV excitation (excitation, 365/12-nm band-pass; emission, 397-nm long-pass).

### Transmission electron microscopy

The ultrastructural changes in the cells of the abscission zone were observed under transmission electron microscopy 8 h after the induction of abscission, and without and with treatment with 1-MCP. One to three pedicels with abscission zones were cross-cut at about 1 mm on the proximal side and about 2 mm on the distal side, with each sampling time as described above. Some samples were cut longitudinally. The samples were fixed with 3% (w/v) glutaraldehyde in 0.08 M phosphate buffer, pH 7.2, post-fixed with 1% (w/v) osmium tetroxide in the same buffer, and embedded in Agar 100 resin (Agar Scientific). Ultrathin sections were stained with 1% (w/v) aqueous uranyl acetate and Reynolds lead citrate. The grids were examined under transmission electron microscope (Philips CM 100), which was operated at an accelerating voltage of 80 kV. The images were recorded using a CCD camera (ORIUS SC 200), and the Digital Micrograph software (Gatan Inc.) was used.

Immunolocalization of ACO1 was compared on ultrathin sections of control pedicels and pedicels at 8 h after abscission induction. The samples were fixed in 4% (w/v) paraformaldehyde and 0.25% (w/v) glutaraldehyde in 0.08 M phosphate buffer for 1 h, and embedded in LR White resin (London Resin Co.). The sections were treated with the ACO antibodies (Santa Cruz Biotechnology, aN-19; 1:100 dilution) for 1 h, and with gold-labeled (10 nm) protein A (Aurion) for 30 min. The control samples were treated only with gold-labeled protein A.

## Results

### The ethylene biosynthesis genes *LeACO1* and *LeACO4* are preferentially expressed in vascular tissues, and are only moderately ethylene sensitive during pedicel abscission

**Figure 1:**
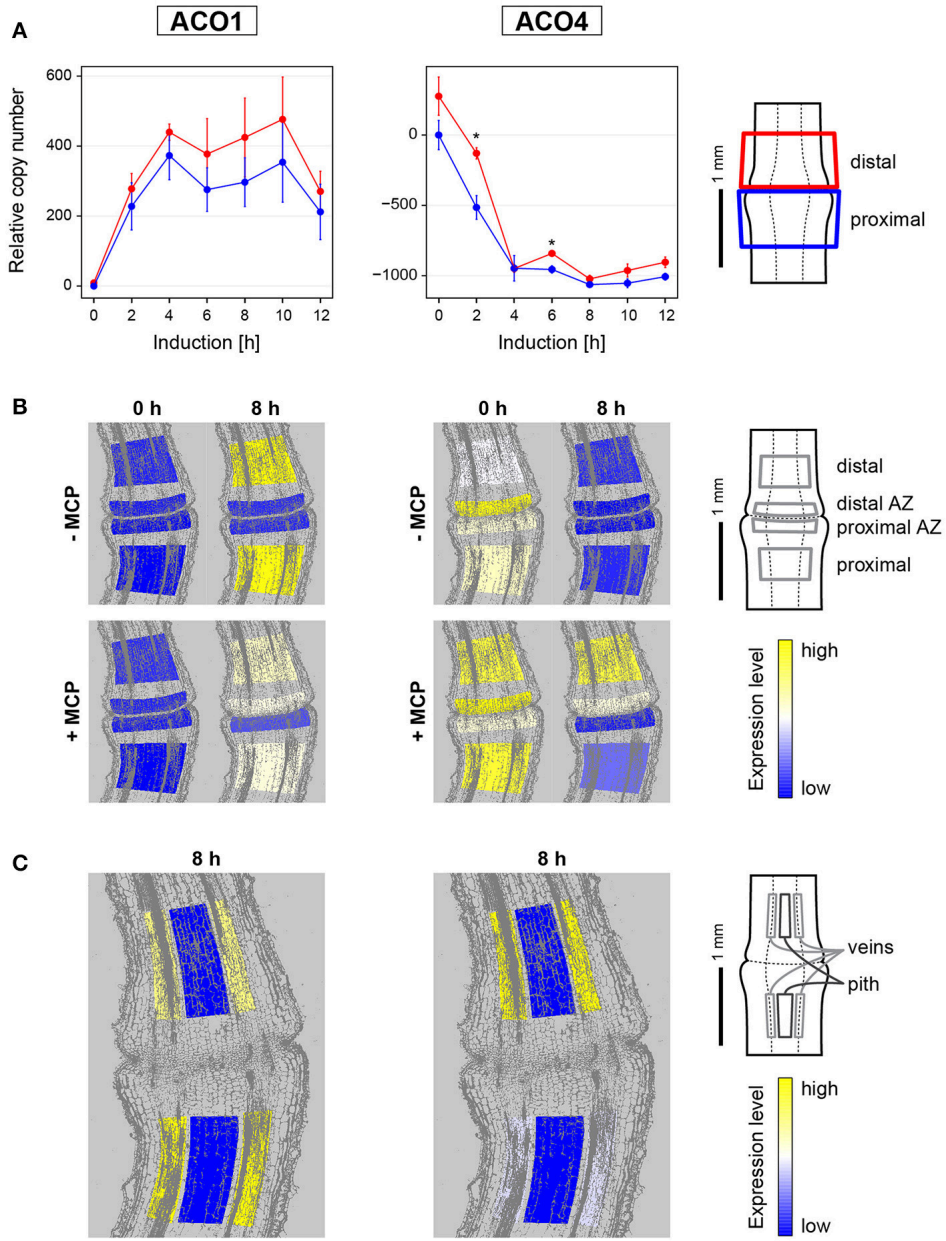
**Gene expression dynamics and tissue specific expression of ethylene synthesis genes ***LeACO1*** and ***LeACO4*** during flower pedicel abscission. (A)** Tomato flower pedicels were sampled before induction (0 h) and 2, 4, 6, 8, 10, and 12 h after induction of abscission, and separated into the proximal and distal sides at the abscission fracture plane, as shown schematically. The data are expressed as relative copy numbers ± SEM from three biological replicates; ^*^p < 0.05), between proximal and distal sides. **(B)** The tomato pedicel region containing the abscission zone (AZ) was divided into four zones using laser microdissection, as shown schematically: distal, distal AZ, proximal AZ, and proximal. Untreated (−MCP) and 1-MCP treated (+MCP) samples were taken before induction (0 h) and 8 h after induction. **(C)** Samples of vascular and pith tissue were analyzed in abscission-induced pedicels. The expression levels for each gene are represented according to a color scale. The exact numeric data are available in the Supplementary Table 3.

The transcription dynamics of two ethylene biosynthetic genes expressed in tomato flower and fruit tissues, *LeACO1* and *LeACO4* (The Tomato Genome Consortium, 2012; Tomato eFP Browser) were followed during pedicel abscission (Figure 1).

Before abscission induction, very low levels of *LeACO1* expression were evenly distributed among the tissues in the abscission zone and in the neighboring proximal and distal tissues (Figure 1). *LeACO1* expression then showed significant changes through the first 12 h after abscission induction (Supplementary Table 2). *LeACO1* expression increased rapidly in the first 4 h, to reach a plateau (Figure 1A), which was maintained until the final separation of the cells in the abscission zone usually 14 h after abscission induction (data not shown). A detailed localization of *LeACO1* transcript in specific cell layers showed that 8 h after abscission induction its level remained very low in the abscission zone, but was high on the proximal and distal sides of it (Figure 1B). *LeACO1* expression was localized mostly in the vascular tissues, with some seen in the pith cells (Figure 1C).

In contrast to *LeACO1*, initial expression levels of *LeACO4* were high (Figure 1). A significant rapid drop in *LeACO4* expression started shortly after abscission induction, and reached a minimum after 4 h (Supplementary Table 2). Although *LeACO4* expression levels followed the same pattern on both sides of the abscission zone, they were still significantly higher distal to it 2 and 6 h after abscission induction (Figure 1A). As for *LeACO1, LeACO*4 was expressed in the vascular tissues (Figure 1C).

To determine whether the expression levels of *LeACO1* and *LeACO4* are susceptible to ethylene-dependent feedback regulation, 1-MCP was applied. The pretreatment of samples with 1-MCP only slightly affected the expression pattern of *LeACO1* and unsignificantly affected the expression pattern of *LeACO4* at the time point 0 h (Figure 1B, Supplementary Figure 4). However, in the presence of this competitive inhibitor of ethylene actions 8 h after abscission induction, *LeACO1* expression levels were slightly lower in the tissues proximal and distal to the abscission zone, in comparison with the untreated samples (Figure 1B). On the contrary, *LeACO1* expression was moderately increased in the distal abscission zone (Figure 1B). The inhibition of *LeACO1* expression by 1-MCP at 8 h after abscission induction was only partial, which is in agreement with a previous report (Meir et al., 2010). On the other hand, 8 h after abscission induction the treatment with 1-MCP prevented the decrease in *LeACO4* expression in the distal abscission zone and in tissues distal to it (Figure 1B).

### ACO1 protein is localized to the phloem

**Figure 2:**
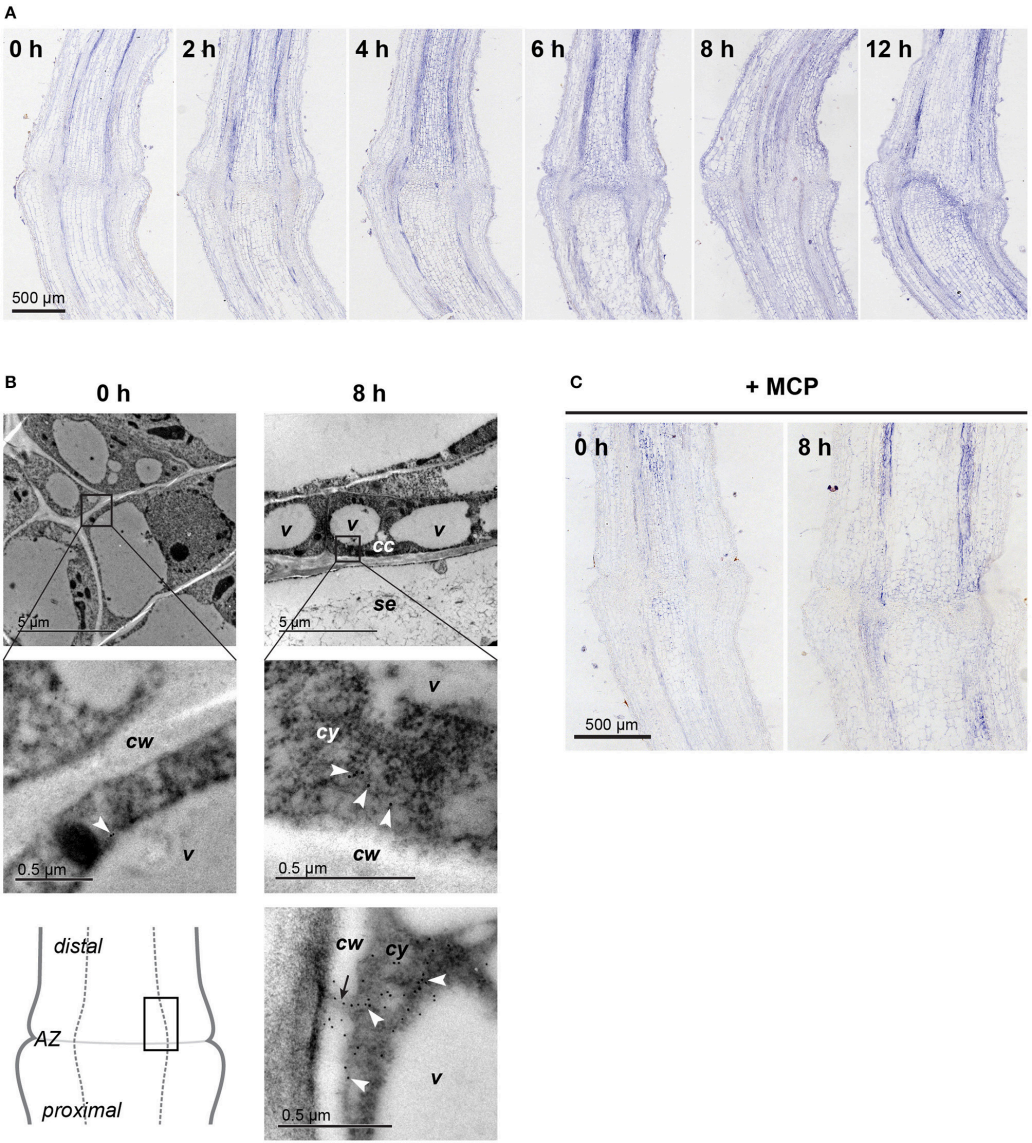
**Localization of the ACO protein in the flower pedicel abscission zone. (A)** Immunolocalization of ACO at 0, 2, 4, 6, 8, and 12 h after induction of abscission, at the light microscopy level. **(B)** Immunogold localization of ACO in the pedicel abscission zone at the transmission electron microscopy level. White arrowheads, gold particles in the cytoplasm; black arrow, plasmodesmata; *cc*, companion cell; *cw*, cell wall; *cy*, cytoplasm; *se*, sieve element; *v*, vacuole. **(C)** Immunolocalization of ACO in 1-MCP–treated pedicels before and 8 h after abscission induction. Negative controls are available in Supplementary Figure 3.

**Figure 3:**
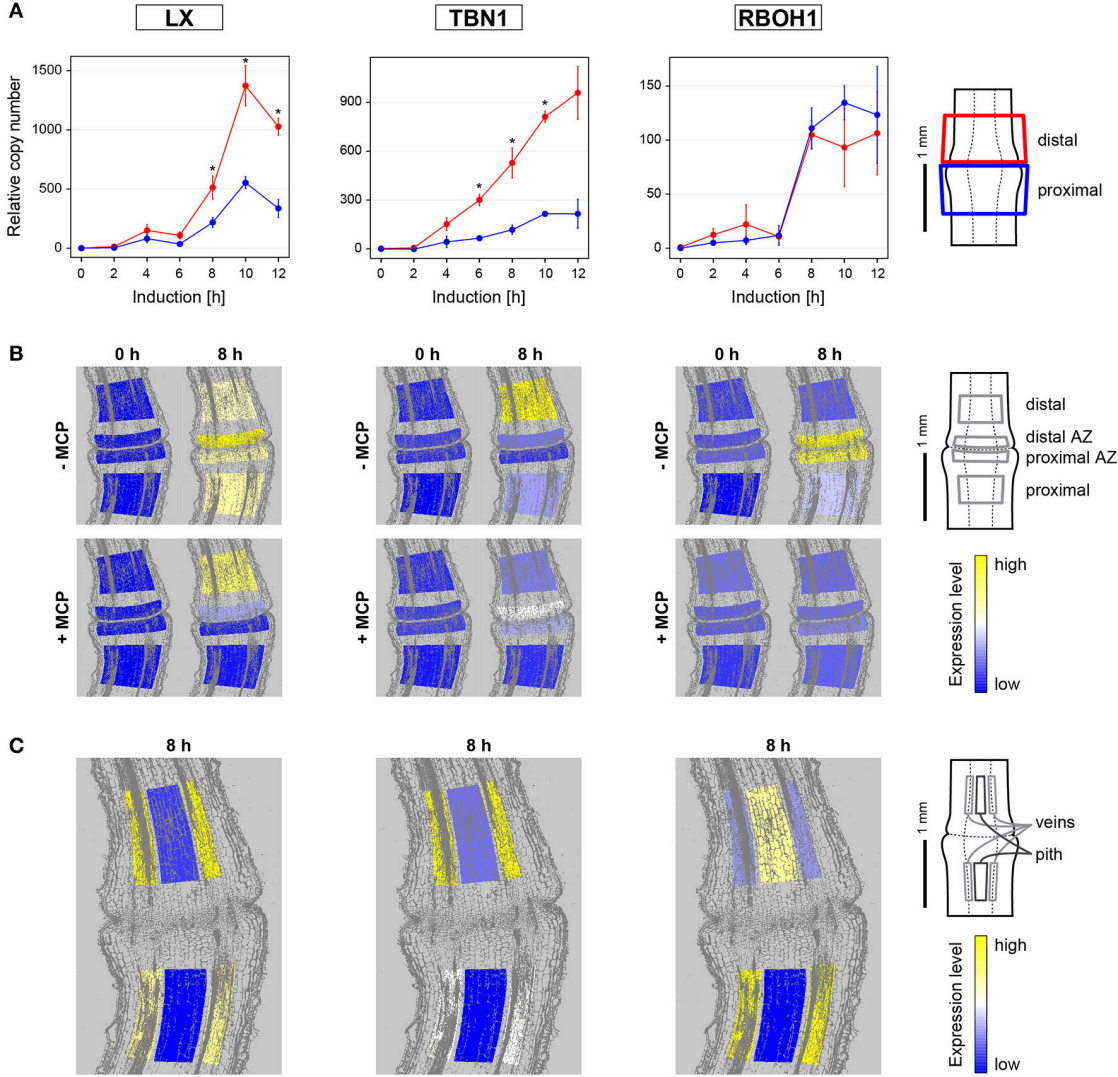
**Gene expression dynamics and tissue specific expression of the PCD-related genes ***LeLX***, ***LeTBN1***, and ***LeRBOH1*** during flower pedicel abscission. (A)** Tomato flower pedicels were sampled 0, 2, 4, 6, 8, 10, and 12 h after induction of abscission, and separated into the proximal and distal sides at the abscission fracture plane, as shown schematically. The data are expressed as relative copy numbers ± SEM from three biological replicates; ^*^p < 0.05, between proximal and distal sides. **(B)** The tomato pedicel region containing the abscission zone (AZ) was divided into four zones using laser microdissection, as shown schematically: distal, distal AZ, proximal AZ and proximal. Untreated (−MCP) and 1-MCP treated (+MCP) samples were taken 0 and 8 h after induction of abscission. **(C)** Samples of vascular and pith tissue were analyzed in abscission-induced pedicels. The expression levels for each gene are represented according to a color scale. The exact numeric data are available in the Supplementary Table 3.

In agreement with the increasing expression of *LeACO1* in the vascular tissues in first 10 h after abscission induction, in the same time period the increasing amount of ACO1 protein was also confirmed to be in the vascular tissues. It was slightly more abundant distal to the abscission zone. (Figure 2A, Supplemenatry Figure 3). The immunolabeling analysis at the subcellular level (Figure 2B) revealed only a few ACO1 gold-labeled particles in the abscission zone vascular tissue before abscission induction. Eight hours after abscission induction, many ACO1 gold-labeled particles specifically appeared in the cytoplasm of the phloem companion cells in the vascular tissues distal to the abscission zone (Figure 2B). A signal was also seen occasionally in the areas of the plasmodesmata and the cell walls (Figure 2B). The pattern of the ACO1 protein accumulation after treatment of the samples with 1-MCP followed that of the of *LeACO1* expression in the corresponding samples (Figure 2C). Specifically, there was a moderate accumulation of ACO1 in the abscission zone as well as in the vascular tissues proximal and distal to it, with slightly stronger ACO1 signal in distal parts of the pedicel.

### The expressions of PCD markers and *LeRBOH1* are differentially affected with the 1-MCP treatment during flower pedicel abscission

*LeLX* expression showed significant asymmetric distribution between the proximal and distal sides of the abscission zone, with prevailing expression on the distal side (Figure 3A, Supplementary Table 2). This increased *LeLX* expression began 6 h after abscission induction, and then *LeLX* expression levels steeply increased over the next 4 h, before dropping after 10 h (Figure 3A). Laser microdissection revealed that the prevailing *LeLX* expression was in the vascular tissues (Figure 3C). The predominant site of *LeLX* expression was in the distal abscission zone (Figures 3A,B). While treatment with 1-MCP completely prevented *LeLX* expression in the pedicel part proximal to the abscission zone and in the proximal abscission zone 8 h after abscission induction, there was less inhibition of *LeLX* expression in the distal abscission zone, and no effects on *LeLX* expression in the part of the pedicel distal to the abscission zone (Figure 3B).

*LeTBN1* expression was significantly asymmetric and changed over time (Figure 3A, Supplementary Table 2). *LeTBN1* expression started to increase 2 h after abscission induction, with a moderate increase in the part of pedicel proximal to the abscission zone over the next 10 h, and a steep and significant increase in the distal part over the same time (Figure 3A). *LeTBN*1 expression in the abscission zone itself was very low (Figure 3B), and it was localized almost exclusively to the vascular tissue (Figure 3B). Of note, treatment with 1-MCP 8 h after abscission induction inhibited the main *LeTBN1* expression in the distal part, and also its very low expression in the proximal part. However, *LeTBN1* expression was even slightly increased in the abscission zone after the 1-MCP application (Figure 3B).

Here we have confirmed our previous report that the expression of the tomato NADPH oxidase gene *LeRBOH1* is specific to the abscission zone tissue (Bar-Dror et al., 2011). However, the laser microdissection revealed no differences in *LeRBOH1* expression between the proximal and distal abscission zone. In addition, *LeRBOH1* was the only gene of those examined where for the distal part, it was localized more to the pith than the vascular tissues (Figure 3B). *LeRBOH1* expression was almost completely prevented with the 1-MCP treatment (Figure 3B).

The pretreatment of samples with 1-MCP before abscission induction did not affect the expression patterns of *LeLX, LeTBN1*, and *LeRBOH1* (Supplementary Figure 4).

### Cell-wall hydrolysing enzymes associated with the flower pedicel abscission are mainly expressed in the narrow abscission zone

**Figure 4:**
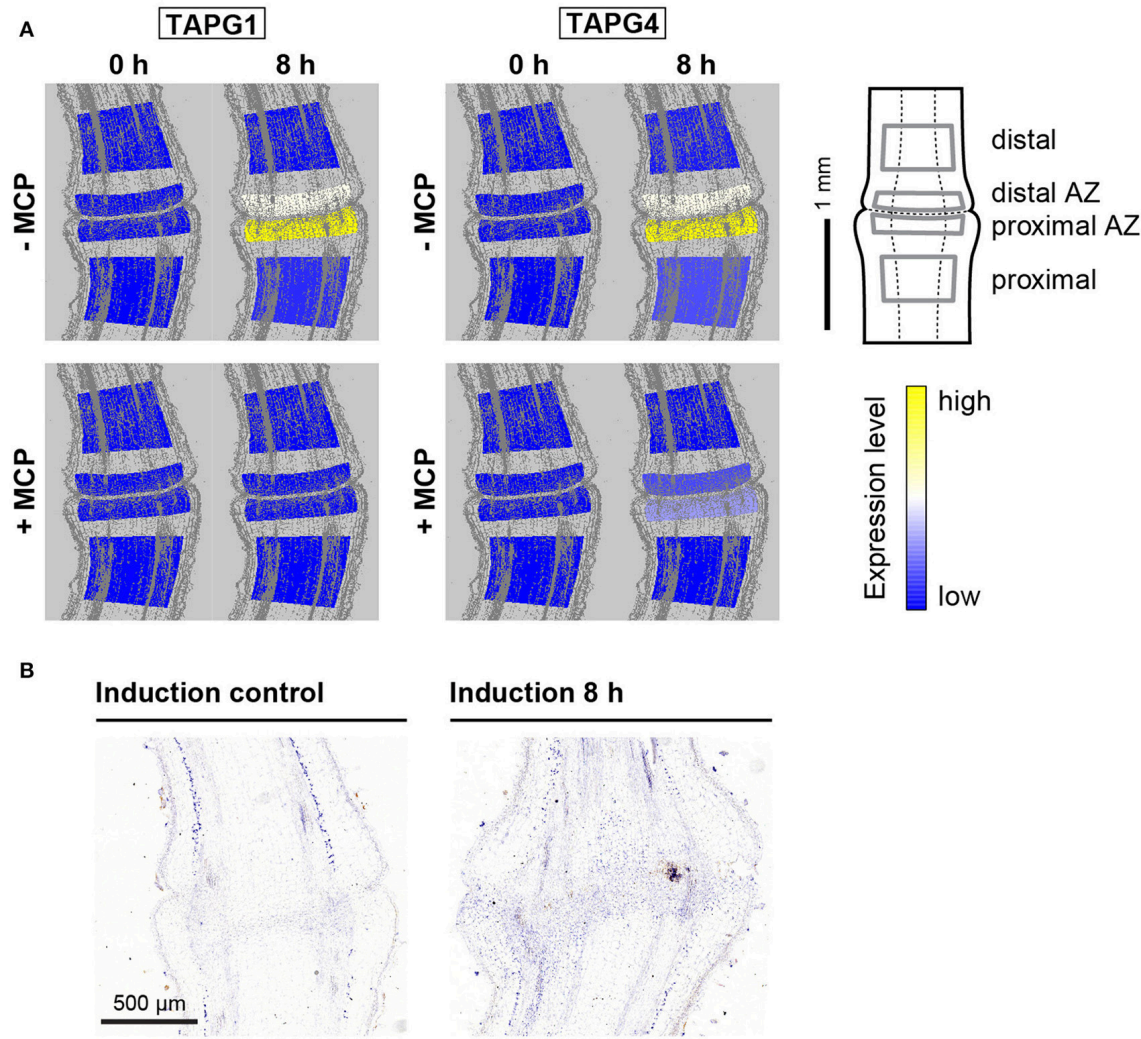
**Tissue-specific expression of polygalacturonase genes and localization of the polygalacturonase protein TAPG4. (A)** The tomato pedicel region containing the abscission zone (AZ) was divided into four zones using laser microdissection, as shown schematically: distal, distal AZ, proximal AZ and proximal. Untreated (−MCP) and 1-MCP treated (+MCP) samples were taken 0 h and 8 h after induction of abscission. The expression levels for each gene are represented according to a color scale. The exact numeric data are available in the Supplementary Table 3 **(B)** Immunolocalization of TAPG4 before and 8 h after abscission induction. Negative controls are available in Supplementary Figure 3.

Expression of the genes that encode the polygalacturonase cell-wall-hydrolysing enzymes *LeTAPG1* and *LeTAPG4* have been reported for the flower abscission zone previously (Kalaitzis et al., 1997; Meir et al., 2010), with their prevalent expression in the pedicel part proximal to the abscission zone (Bar-Dror et al., 2011). *LeTAPG1* and *LeTAPG4* expression were almost completely prevented by 1-MCP (Meir et al., 2010). Here, *LeTAPG1* and *LeTAPG4* expression were almost completely limited to the abscission zone, with slightly higher expression in the proximal abscission zone (Figure 4A). Of note, while 1-MCP treatment completely inhibited the *LeTAPG1*expression, there was still some remaining *LeTAPG4* expression in the abscission zone (Figure 4A). Immunolocalization using antibodies against TAPG4 confirmed these findings at the protein level (Figure 4B, Supplementary Figure 3).

### Cellular morphology and DNA fragmentation are differentially affected by 1-MCP during the pedicel abscission induction

**Figure 5:**
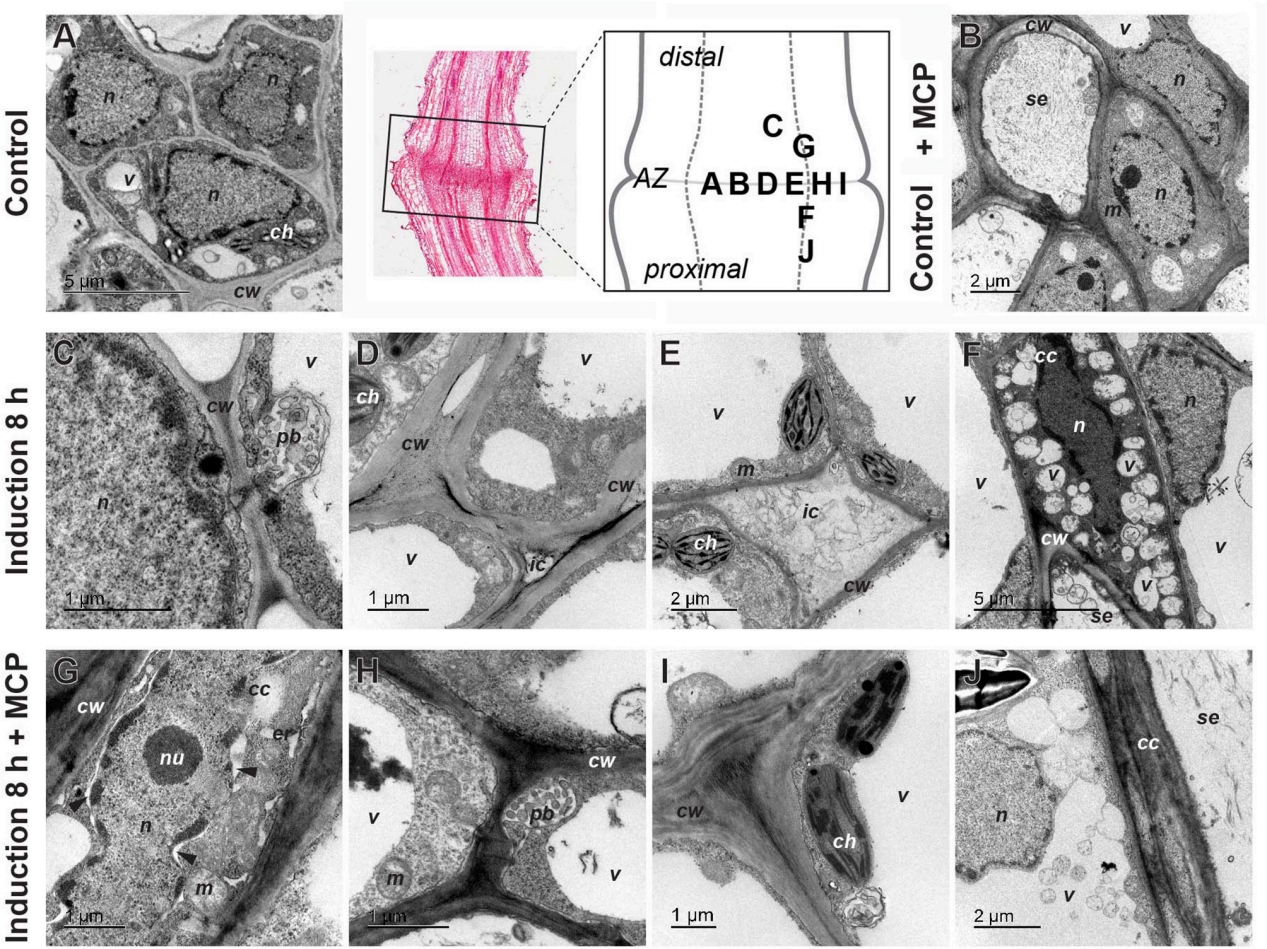
**Ultrastructure of tomato flower pedicel cells before induction of abscission (A)**, before induction of abscission and pretreated with 1-MCP **(B)**, 8 h after induction **(C–F)** and 8 h after induction and 1-MCP treatment **(F–J)**. **(A,B)** The cells in the abscission zone (AZ) before abscission induction were small and tightly packed. **(C)** Formation of the paramural body. **(D)** Appearance of changes in the cell wall and the beginning of cell separation. **(E)** Intercellular spaces filled with nonhomogeneous material. **(F)** Nucleus in companion cell with partly condensed chromatin, in contrast to nucleus in adjacent cell and small vacuoles/vesicles filled with material. **(G)** Nucleus with partly condensed chromatin, dilated nuclear membrane (arrowheads), and dilated endoplasmic reticulum, in a companion cell. **(H)** Paramural body. **(I)** Nonseparated cells with some changes in cell-wall structure. **(J)** Fusion of small vacuoles into a large one. The part of the pedicel that was used to prepare sections is indicated by a rectangle in the histological section and the letters in the schematic abscission zone drawing indicate the places from where the individual micrographs were taken (Control lane, middle). *cc*, companion cell; *ch*, chloroplast; *cw*, cell wall; *er*, endoplasmic reticulum; *ic*, intercellular space; *m*, mitochondrion; *n*, nucleus; *nu*, nucleolus; *pb*, paramural body; *se*, sieve tube element; *v*, vacuole/vesicle.

Using transmission electron microscopy, the ultrastructural changes in the abscission zone were examined before induction of abscission and 8 h afterwards (Figure 5). Before induction, differentiated cells in the pedicel abscission zone were morphologically and physiologically distinct from the neighboring cells (Figure 5A). They resembled meristematic cells, and indeed, some of the genes expressed in the abscission zone have been shown to be associated with organ differentiation in meristems (Nakano and Ito, 2013; Wang et al., 2013; Kim et al., 2016). These cells were small and tightly packed together, without intercellular spaces. They had dense cytoplasm, with small vacuoles. Their nuclei were round to oval, and their chloroplasts were smaller (i.e., around 1.9 × 0.9 μm), in comparison to the chloroplasts in the pedicel cortex outside the abscission zone (i.e., 4.0 × 2.2 μm). The plasma membrane was tightly fitted to the cell wall. The nuclei of the sieve element companion cells in the phloem were similar to those in the other abscission zone cells, with heterochromatin at the border. Sieve tube elements contained endoplasmic reticulum and mitochondria near the plasma membrane, and in some regions, filaments of P-proteins were seen (Supplementary Figure 5).

The abscission always started in the companion cells in the central abscission zone. One of the first signs of abscission was detachment of the plasma membrane from the cell wall (Figures 5C–E). Also early in the process, many vesicle-like small vacuoles developed in the peripheral cytoplasm of the cells (Figure 5F). These were more abundant in the abscission zone and the tissues proximal to it, and they were commonly packed with deposits. In addition to these small vacuoles, the cells in the area of abscission were also characterized by different kinds of vesicles, which were often packed in paramural bodies between the plasma membrane and the cell wall (Figure 5C). The nuclei of many cells in the area became electron dense (Figure 5F). In some cells, dilatation of the endoplasmic reticulum was observed (Supplementary Figure 5D). At this stage of abscission, the intercellular space was greatly increased between the cells in the abscission zone, and it commonly became densely filled with material (Figures 5D,E).

To examine the association of the observed changes during pedicel abscission induced by ethylene, the plants were again treated with 1-MCP. The pretreatment with 1-MCP of samples did not significantly affect the morphology of the abscission zone at time 0 h (Figure 5B). However, 8 h after abscission induction, this treatment had profound effects specifically on the separation of the cells. The cells stayed packed tightly together, and in general, there was no enlargement of the intercellular space, although there were some changes in the cell wall structure (Figures 5H,I). On the other hand, the 1-MCP treatment did not affect the formation of membrane evaginations, and the formation of multivesicular and paramural bodies, as well as small vacuoles, which appeared at similar quantities as in the 1-MCP untreated samples (Figures 5H,I). In addition, features associated with PCD in the distal part of abscission zone were not affected by the 1-MCP treatment. Several cells had plasma membranes detached from the cell wall, dilated ER, and nuclei with condensed chromatin and dilated nuclear membrane (Figure 5G). Cells with similar characteristics also appeared in the proximal part of abscission zone, but their frequency was low.

**Figure 6:**
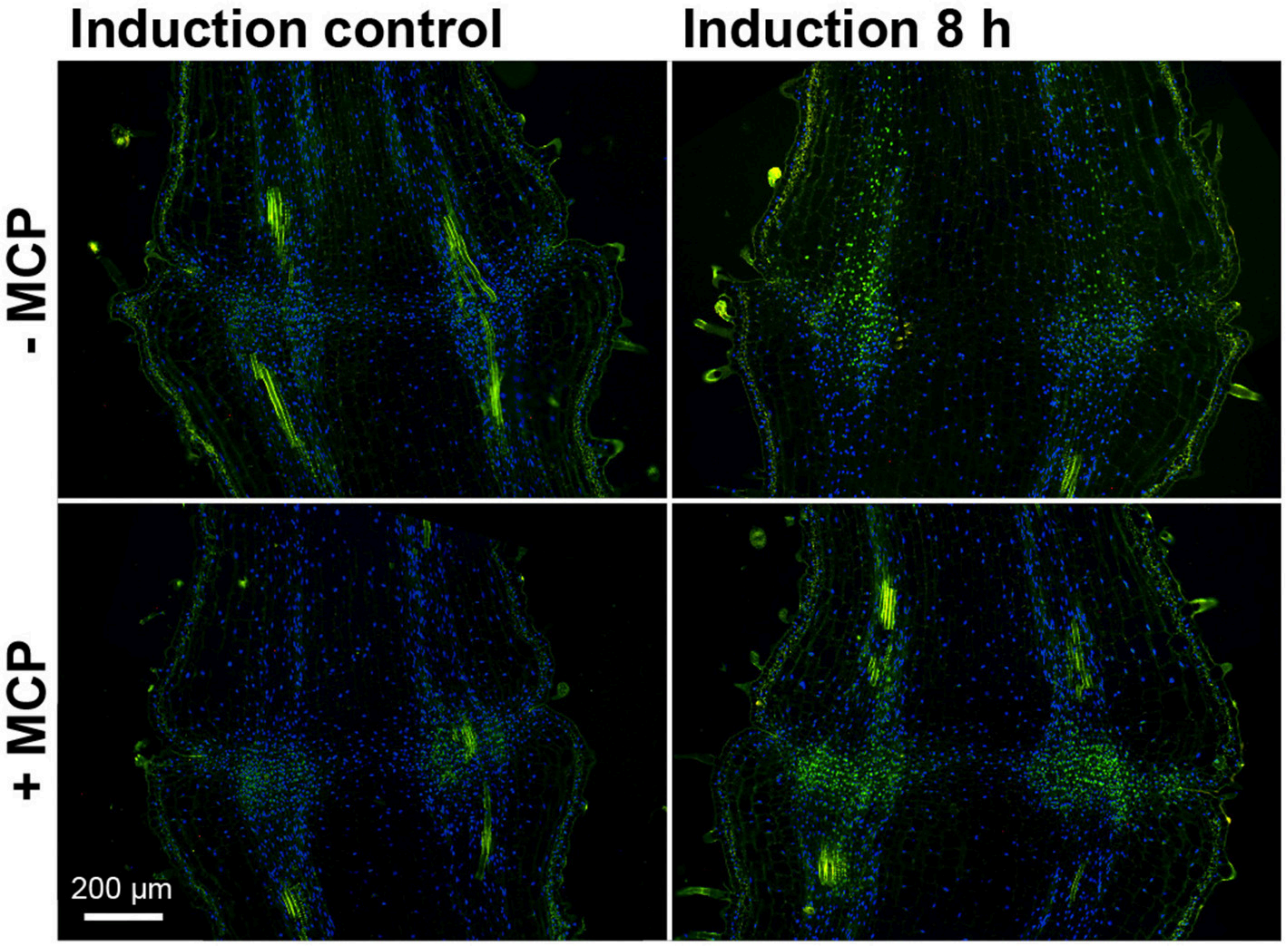
**DNA fragmentation in the tomato flower pedicel abscission zone**. Tissue sections of 1-MCP untreated (−MCP) and treated (+MCP) samples taken at 0 h (induction control) and 8 h after induction of abscission, and stained for the TUNEL assays. Green, TUNEL-positive nuclei with fragmented DNA; blue, nuclei with non-fragmented DNA (DAPI staining). The green signal outside the nuclei is due to autofluorescence of the cell walls. Images are composites of two fluorescence channels.

To follow PCD at the cellular level, TUNEL assays were used that selectively stain damaged DNA. Eight hours after abscission induction, the TUNEL assays showed prevalent positive fluorescence staining of the nuclei along the vascular tissue toward the pedicel part distal to the abscission zone (Figure 6). After treatment with 1-MCP, there was a strong green signal that was almost completely limited to the abscission zone.

## Discussion

### ACO1 is synthesized in the phloem

The generally accepted model of abscission induction in tomato involves reduction in auxin level and induction of ethylene production and ethylene signaling (Taylor and Whitelaw, 2001; Meir et al., 2010, 2015; Nakano and Ito, 2013). However, the location where the last enzyme in the ethylene biosynthetic pathway, ACO, is synthesized during abscission is demonstrated here for the first time to be just outside the abscission zone. The expression of both of the flower pedicel *ACO* genes, i.e., *L*e*ACO1* and *LeACO4*, occurred almost exclusively in the vascular tissues.

Previous data relating to the intracellular localization of ACO protein has been contradictory. While studies of suspension-cultured tomato cells and apple fruit pericarp tissue showed ACO in the cytoplasm (Reinhardt et al., 1994; Chung et al., 2002), immune-cytolabeling of apple fruit protoplast saw ACO located at the external side of the plasma membrane (Ramassamy et al., 1998). However, the present study offers support to all of these. In accordance with the *LeACO1* expression pattern the majority of ACO1 protein was in the phloem-cell cytoplasm, although some signal was seen in the area of the phloem-cell plasmodesmata and the cell walls.

As ACO is the last step in ethylene biosynthesis, it is rational to predict from our results that ethylene biosynthesis takes place in the phloem, from where it diffuses to the other tissues in order to act. It remains enigmatic if and how this is related to several suggestions of diffusible signals derived from vascular tissue that are required for abscission (Thompson and Osborne, 1994; McManus, 2008; Tucker and Yang, 2012).

The present findings combined with some of the literature data (Meir et al., 2010) indicate that pedicel abscission follows the broadly accepted concept of two systems of ethylene biosynthesis described during climacteric fruit development and ripening (McMurchie et al., 1972; Van de Poel et al., 2012). As a part of system 1, the *ACO4* expression before abscission induction was high and it decreased simultaneously with the inducing high expression of *LeACO1* (present study) and *ACO5* expression (Meir et al., 2010) in system 2, at 2 h after the abscission induction. Similar expression pattern of the *ACO* genes has also been demonstrated in the leaf petiole abscission zone (Chersicola et al., in review). The specific temporal distribution of *ACO* genes is also reflected in their spatial localization inside the abscission zone and in its close vicinity. While the initial high level of *LeACO4* expression prevailed in the distal part of the abscission zone, *LeACO1* expression was exclusively in cell layers in both sides of the pedicel outside the abscission zone. While this might appear to contradict the recently published transcriptome data of Kim et al. (2015) that show *LeACO1* expression in the pedicel abscission zone, Kim et al. (2015) also pointed out that collection of abscission data is not uniform across all studies. Accordingly, after microdissection, *LeACO1* expression was seen outside the abscission zone specified in the present study, although inside the abscission zone area defined by Kim et al. (2015). On the other hand, *LeACO4* expression before abscission induction was more synchronized between these two studies, due to abundant *LeACO4* expression inside the abscission zone (this study; Kim et al., 2015).

Of note, after the treatment with the competitive inhibitor of ethylene actions, 1-MCP, the decrease of *LeACO4* expression that was seen in the untreated samples 8 h after abscission induction, was prevented in the distal abscission zone as well as in tissues distal to it. At the same time, *LeACO1* expression pattern was only partially affected in most of the tissues examined, with slightly induced levels in the distal part of the abscission zone. These data complement a previous microarray study (Meir et al., 2010), and suggest that during tomato pedicel abscission, *LeACO1* expression is developmentally regulated and is not completely dependent on ethylene-related signaling.

### Cell separation and PCD are differentially sensitive to ethylene

To test the hypothesis that the underlying regulatory mechanism for the abscission processes is based on ethylene signaling, we followed the specific expression of the genes in the abscission-zone tissue after the treatment of the samples with 1-MCP. The *TAPG4, TAPG1, Cel1*, and *Cel5* genes that encode cell-wall hydrolyzing enzymes are almost completely inhibited by 1-MCP (this study; Meir et al., 2010). As has also been shown previously, mRNA of the polygalacturonase-encoding gene *TAPG4* is detected much earlier than *TAPG1* mRNA (Kalaitzis et al., 1997; Meir et al., 2010; Bar-Dror et al., 2011) and *Cel1* and *Cel5* expression, which encode cellulases (Meir et al., 2010; Bar-Dror et al., 2011). Additionally, the present study shows expression of the TAPG4 protein in the proximal abscission zone. The ultrastructural images of the flower pedicel abscission zone at 8 h from abscission induction support these data, as they showed the increased intercellular space filled with deposits. At the same time after abscission induction, formation of the intercellular space was completely prevented in the samples treated with 1-MCP. It thus appears reasonable to assume that formation of the intercellular space at 8 h after abscission induction is a result of polygalacturonase activity, and that other cell-wall hydrolyzing enzymes are needed for the completion of abscission.

On the other hand, the sensitivity to ethylene of the PCD marker genes *LeLX* and *LeTBN1* (Olvera-Carrillo et al., 2015) and *LeRBOH1* depends on the gene location in the specific cell layer inside the abscission zone, or in its vicinity. Nevertheless, 1-MCP had stronger effects on PCD-related gene expression in cells in distal abscission zone. Although this was associated with more cells with damaged DNA inside the abscission zone, as confirmed by the TUNEL assays, 1-MCP treatment did not completely inhibit PCD at the ultrastructural level. The temporal profile of *LeRBOH1* expression and the vascular tissue localization of this concurred with that of *LeLX* expression, and also roughly of *LeTBN1* expression. However, *LeRBOH1* expression was limited to the abscission zone, and this was substantially inhibited by 1-MCP. It remains under debate whether the NADPH-oxidase encoded by *LeRBOH1* and the generation of superoxide on the apoplastic side are crucial to the triggering of developmental PCD (De Pinto et al., 2012). However, the spatio-temporal pattern of *LeRBOH1* expression during flower abscission, and the similar time for induction of *LeRBOH1* expression, appear to support the suggestion of its involvement in PCD during abscission.

An interesting observation at the subcellular level was the formation of the paramural bodies between the plasma membrane and the cell wall, which was not affected by 1-MCP treatment. Similar structures have been reported for the leaf abscission zone that were strictly associated with the proximal side of the abscission-zone fracture (Bar-Dror et al., 2011; Dermastia et al., 2012). It has been suggested that these vesicles have a role during the development of a protective layer at the surface of the exposed tissue, following the release of the cells at the site of leaf detachment from the plant body (Bar-Dror et al., 2011; Dermastia et al., 2012). Similar structures have also been observed in Arabidopsis mutants, in which the shedding of the floral organ can be blocked by disruption of the function of the ADP-ribosylation factor and GTPase-activating protein NEVERSHED (NEV) (Liljegren et al., 2009). Thus, it has been suggested that NEV activity is required for correct trafficking of cargo molecules during cell separation, and that the formation of paramural bodies is associated with trafficking defects (Liljegren et al., 2009). Although the role of NEV homologs has not been explored in tomato, the studies on tomato flower pedicel and leaf petiole abscission (this study; Bar-Dror et al., 2011) indicate that the formation of paramural bodies is an essential part of organ abscission in this plant.

### The perpendicular spread of the flower pedicel abscission processes

**Figure 7:**
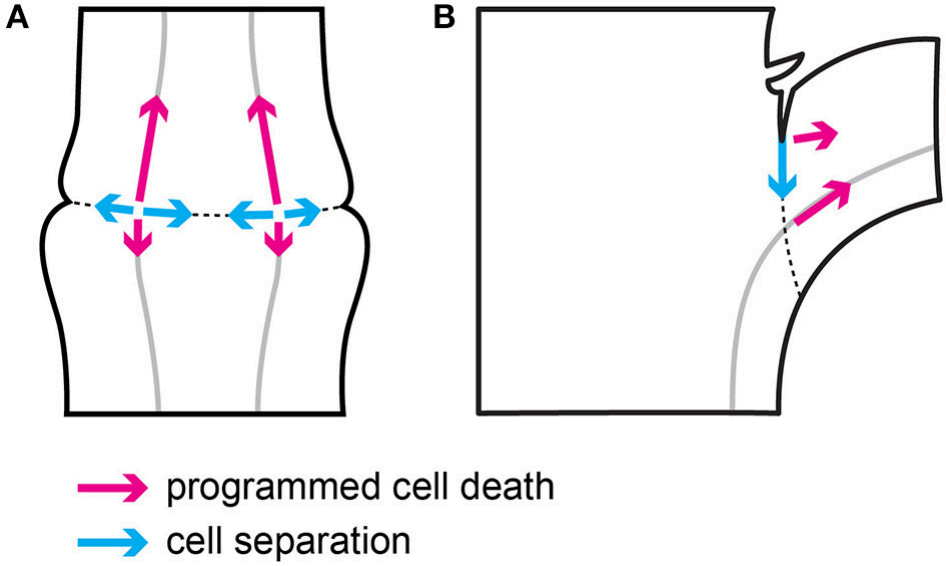
**Schematic representation of the direction of cell separation and PCD during flower pedicel (A)** and leaf petiole **(B)** abscission.

The results of this study show that two main processes involved in tomato flower pedicel abscission—cell separation and PCD (Bar-Dror et al., 2011) share a common origin in the vascular tissues inside the abscission zone, although they progress in perpendicular directions in terms of their spread as shown schematically in Figure 7A.

The cell separation in the flower pedicel abscission zone starts in the phloem companion cells in vascular tissues that cross the swollen node, which defines the abscission zone. From there, the cell separation spreads along a very narrow line of cell layers toward both of the edges of the abscission zone (Figure 7A). This cell separation progression follows the temporal expression pattern of the two polygalacturonase genes *LeTAPG1* and *LeTAPG4* (Kalaitzis et al., 1997; Meir et al., 2010; Bar-Dror et al., 2011), which encode the main cell-wall-hydrolyzing polygalacturonase enzymes that are responsible for the dissolving of the middle lamellae that keep cells together. Both *LeTAPG1* and *LeTAPG4* expression and the TAPG4 protein were limited to the flower pedicel abscission zone, with their prevailing expression at the proximal side of the abscission zone.

On the other hand, the morphological hallmarks of PCD are seen to spread from their start in the vascular tissues in the abscission zone, to progress gradually along the vessels, preferentially to the distal side of the abscission zone, and further out distal of the abscission zone (Figure 7A). In agreement with this, the prevailing expression of two PCD marker genes, *LeLX* and *LeTBN1* (Olvera-Carrillo et al., 2015), was in the vascular tissues in the distal abscission zone and distal to it. Of note, during the abscission of both flowers (the present study) and leaves (Bar-Dror et al., 2011), PCD spreads perpendicular to cell separation (Figure 7). Moreover, in both processes the signal for PCD initiation arises from the vascular tissues, as shown by the gene expression and TUNEL analysis (the present study; Bar-Dror et al., 2011).

## Concluding remarks

The outcomes of this study show that changes occurring during tomato flower pedicel abscission are cell-layer-specific on the spatiotemporal basis. The temporal expression pattern of *LeACO1* and *LeACO4*, encoding the last enzyme in the ethylene biosynthetic pathway, follows the concept of two systems of ethylene biosynthesis that has been described during climacteric fruit development and ripening. In accordance with the site of *LeACO1* gene expression, the encoded protein is localized mainly to the cytoplasm of the phloem companion cells just outside the abscission zone. These results suggest that the abscission induced ethylene synthesis also occurs in vascular tissues out of the abscission zone. The inhibition of ethylene action with 1-MCP prevented the separation of cells in the abscission zone, likely by inhibition of cell-wall hydrolyzing enzymes. On the other hand, 1-MCP has only moderate effects on the expression of two marker genes of PCD, *LeLX* and *LeTBN1* and the appearance of the hallmarks of PCD. In addition, the formation of paramural bodies seems to be an important part of abscission in tomato. Although the reason for differential sensitivity of different gene expression in different cell layers shown in this study cannot be explained at the moment, differential effects of 1-MCP to gene expressions in different cell layers might indicate a possible combination of several regulatory steps. This suggestion could also include the expression pattern of *LeEIL2* (Supplementary Figure 6), an ortholog of the Arabidopsis *EIN3* gene in tomato. In Arabidopsis, the binding of the transcription factor EIN3, which controls a multitude of downstream transcriptional cascades, includes a major negative feedback loop (Chang et al., 2013). Taking together, the cell-layer-specific spatiotemporal patterns of abscission events and their responsiveness to ethylene may have an applicable value for fine-tuning of the timing of abscission in alternative strategies for preventing preharvest fruit drop and for extending the lifetime of cut flowers or for promoting early abscission of buds and fruits to stimulate more vigorous plant growth and higher quality of fruits and flowers.

## Author contributions

MC participated in the design of the study, drafted the manuscript, and performed the gene expression analysis; AK participated in the design of the study, preparation of samples and performance of TUNEL, immunolocalization experiments and writing of the manuscript; MT performed the TEM analysis; TM participated in preparation of the laser microdissection slides; KG helped with bioinformatics analysis, and MD participated in the design of the study, coordinated the study, and finalized the manuscript.

### Conflict of interest statement

The authors declare that the research was conducted in the absence of any commercial or financial relationships that could be construed as a potential conflict of interest.

## Footnotes

^1^Tomato eFP Browser Rose Lab Atlas. Available online at: http://bar.utoronto.ca/efp_tomato/cgi-bin/efpWeb.cgi (Accessed August 23, 2016).
