# Supplementary material for: 1-Aminocyclopropane-1-Carboxylate Oxidase Induction in Tomato Flower Pedicel Phloem and Abscission Related Processes Are Differentially Sensitive to Ethylene
Source: Front Plant Sci. 2017 Mar 31;8:464. doi: 10.3389/fpls.2017.00464 (PMC5374216; doi:10.3389/fpls.2017.00464)
Supplement: Supplementary file 1 [file Image1.PDF]

1-aminocyclopropane-1-carboxylate oxidase induction in tomato flower pedicel phloem and abscission related processes are differentially sensitive to ethylene

Marko Chersicola, Aleš Kladnik, Magda Tušek Žnidarič, Tanja Mrak, Kristina Gruden, Marina Dermastia.
Correspondence: marina.dermastia@nib.si

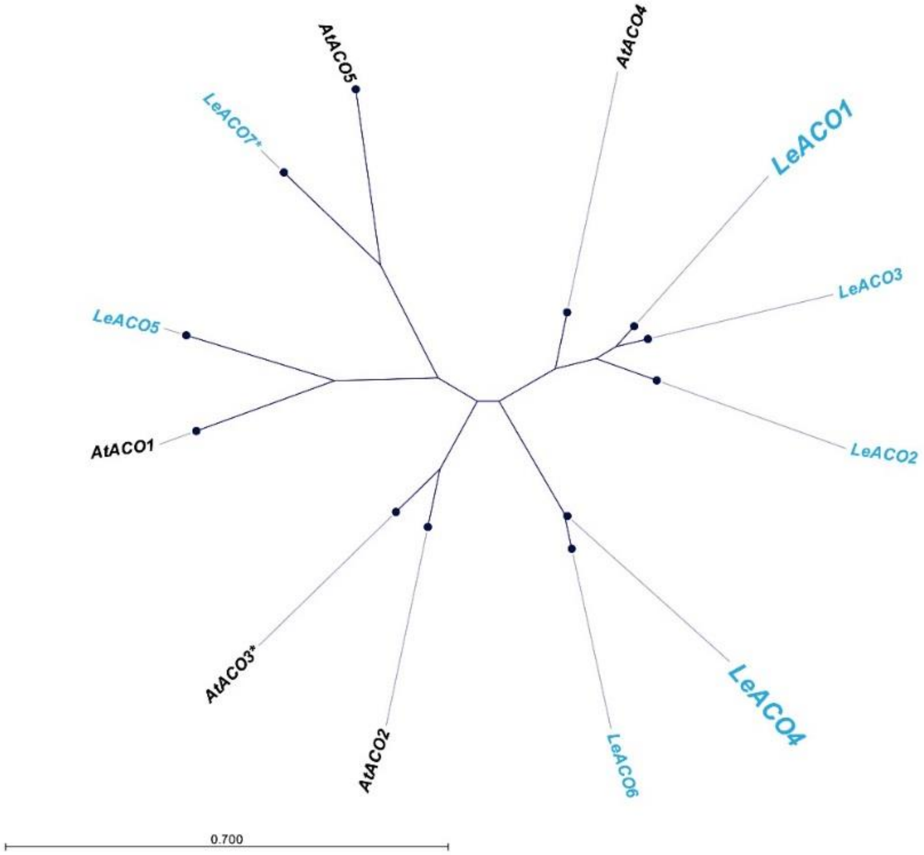

Supplementary Figure S1. Maximum likelihood phylogenetic tree for the *Arabidopsis thaliana* and tomato (*Solanum lycopersicum*) ACO genes. Using CLC Main Workbench software version 6.8.3 (Qiagen), multiple sequence alignment was produced from five *Arabidopsis ACO* genes: AT2G19590, ***AtACO1***; AT1G62380, ***AtACO2***; AT1G12010, ***AtACO3****; AT1G05010, ***AtACO4***; AT1G77330, ***AtACO5****; and seven tomato *ACO* genes: Solyc07g049530, ***LeACO1***; Solyc12g005940, ***LeACO2***; Solyc07g049550, ***LeACO3***;

Solyc02g081190, ***LeACO4***; Solyc07g026650, ***LeACO5***; Solyc02g036350, ***LeACO6***; Solyc06g060070, ***LeACO7**** using the MUSCLE algorithm. An unrooted phylogenetic tree was constructed from the nucleotide multiple sequence alignment, again using CLC Main Workbench software version 6.8.3 (Qiagen), with the following parameters: Starting Tree = Neighbor Joining; Substitution rate model = Jukes Cantor; Include rate variation = Yes; Number of substitution rate categories = 4; Initial Gamma distribution parameter = 1.0; Estimate Gamma distribution parameter = Yes; Estimate substitution rate parameter(s) = Yes; and Estimate topology = Yes. The branch lengths represent the number of nucleotide substitutions per site. Gene names in the tree that are marked with an asterisk (*) are given as there is no other reference in the databases or the literature.
