# Supplementary material for: 1-Aminocyclopropane-1-Carboxylate Oxidase Induction in Tomato Flower Pedicel Phloem and Abscission Related Processes Are Differentially Sensitive to Ethylene
Source: Front Plant Sci. 2017 Mar 31;8:464. doi: 10.3389/fpls.2017.00464 (PMC5374216; doi:10.3389/fpls.2017.00464)
Supplement: Supplementary file 5 [file Image5.PDF]

1-aminocyclopropane-1-carboxylate oxidase induction in tomato flower pedicel phloem and abscission related processes are differentially sensitive to ethylene

Marko Chersicola, Aleš Kladnik, Magda Tušek Žnidarič, Tanja Mrak, Kristina Gruden, Marina Dermastia.
Correspondence: marina.dermastia@nib.si

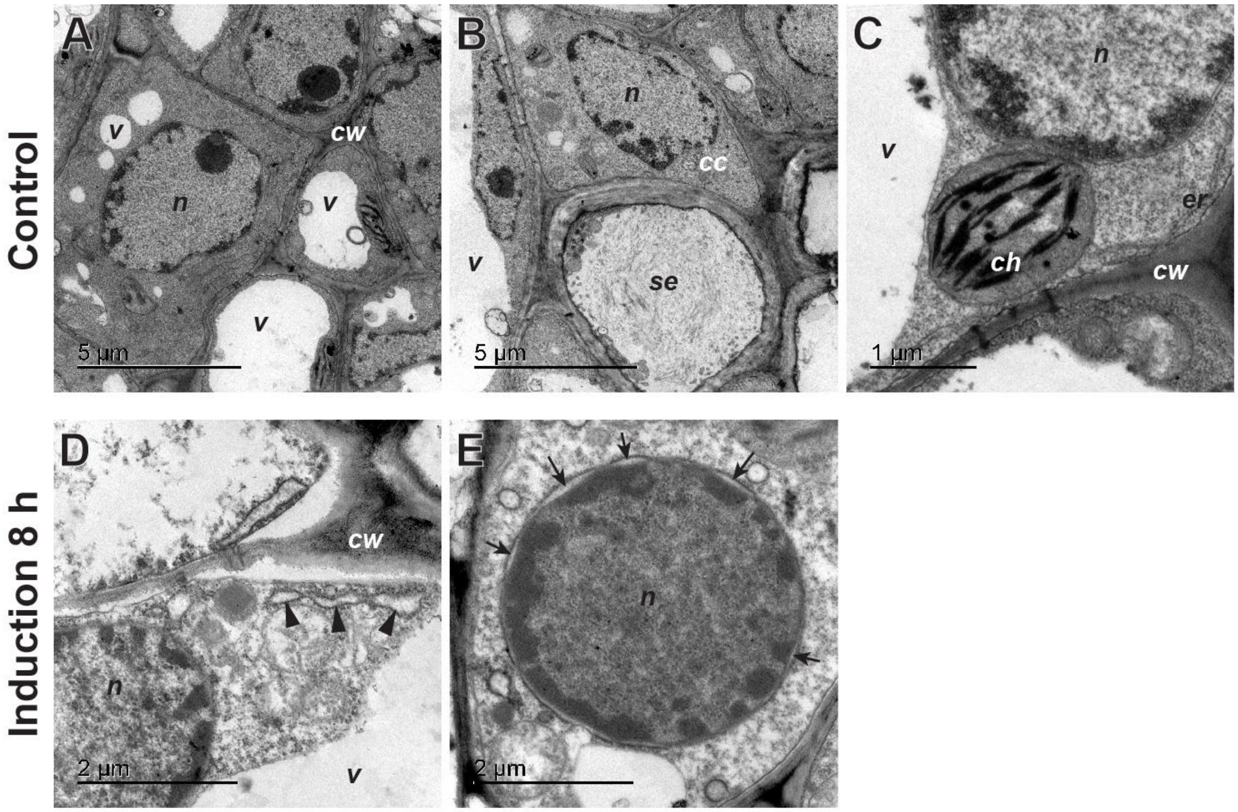

Supplementary Figure S5. Ultrastructure of tomato flower pedicel cells before the induction of abscission (Control) and 8 h after abscission induction (Induction 8 h). (A) Cells of abscission zone with dense cytoplasm and smaller vacuoles; plasmalemma tigtly fit to the cell wall; **(B)** vascular tissue in the abscission zone; sieve tube element with endoplasmic reticulum and mitochondria at the border; **(C)** higher magnification of the cell in the abscission zone with dense cytoplasm, chloroplast and part of nucleus; **(D)** dilatation of endoplasmic reticulum (black arrowheads) in the cell of abscission zone after abscission induction; **(E)** dilatation of nuclear membranes (black arrows) and beginning of chromatin condensation in the cell of abscission zone after abscission induction; *cc*, companion cell; *ch*, chloroplast; *cw*, cell wall; *er*, endoplasmic reticulum; *n*, nucleus; *se*, sieve tube element; *v*, vacuole.
