# Supplementary material for: 1-Aminocyclopropane-1-Carboxylate Oxidase Induction in Tomato Flower Pedicel Phloem and Abscission Related Processes Are Differentially Sensitive to Ethylene
Source: Front Plant Sci. 2017 Mar 31;8:464. doi: 10.3389/fpls.2017.00464 (PMC5374216; doi:10.3389/fpls.2017.00464)
Supplement: Supplementary file 6 [file Image6.PDF]

1-aminocyclopropane-1-carboxylate oxidase induction in tomato flower pedicel phloem and abscission related processes are differentially sensitive to ethylene

Marko Chersicola, Aleš Kladnik, Magda Tušek Žnidarič, Tanja Mrak, Kristina Gruden, Marina Dermastia.
Correspondence: marina.dermastia@nib.si

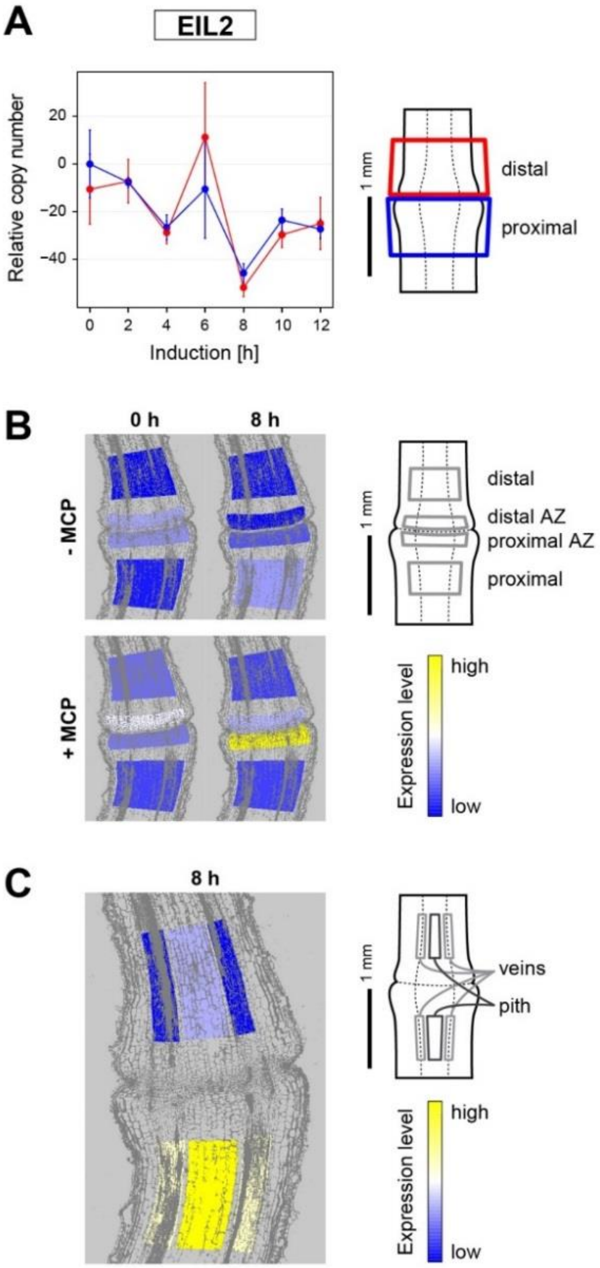

Supplementary Figure S6. Relative gene expression of *LeEIL2*. (A) Tomato flower pedicels were sampled before induction (0 h) and 2, 4, 6, 8, 10 and 12 h after induction of abscission, and separated into the proximal and distal sides at the abscission fracture plane, as shown schematically. The data are expressed as relative copy numbers ±SEM from three biological replicates; *, $p < 0.05$), between proximal and distal sides. (B) The tomato pedicel region containing the abscission zone (AZ) was divided into four zones using laser microdissection, as shown schematically: distal, distal AZ, proximal AZ and proximal. Untreated (-MCP) and 1-MCP treated (+MCP) samples were taken before induction (0 h) and 8 h after induction. (C) Samples of vascular and pith tissue were analyzed in abscission induced pedicels. The expression levels for each gene are represented according to a color scale. The exact numeric data are available in the Supplementary Table S3.
