# Supplementary material for: 1-Aminocyclopropane-1-Carboxylate Oxidase Induction in Tomato Flower Pedicel Phloem and Abscission Related Processes Are Differentially Sensitive to Ethylene
Source: Front Plant Sci. 2017 Mar 31;8:464. doi: 10.3389/fpls.2017.00464 (PMC5374216; doi:10.3389/fpls.2017.00464)
Supplement: Supplementary file 7 [file Table1.PDF]

1-aminocyclopropane-1-carboxylate oxidase induction in tomato flower pedicel phloem and abscission related processes are differentially sensitive to ethylene

Marko Chersicola, Aleš Kladnik, Magda Tušek Žnidarič, Tanja Mrak, Kristina Gruden, Marina Dermastia.
Correspondence: marina.dermastia@nib.si

Supplementary Table S1. List of primer and probe sequences used in this study.

Gene	Forward primer	Reverse primer	Probe	ITAG Identifier
LeACO1	AAGGGACTCCGCGCTCATA	CAAGTTGGTCACCAAGGTTAACC	TCGATGTTCCTCCCATGCGCC	Solyc07g049530
LeACO4	GGGAGAGCACTTTCTTCTTGAAACA	CTTCAGTGCAAAATCTTTCATAACTTTCCT	CAGGTCAGGAACTTCA	Solyc02g081190
LeEIL2	GTGTCTTCATTGCCCTCATAGTGA	TGATTGTCTCTGCTGGATCTGTCT	TTCGCAATGGTTTTCC	Solyc01g009170
LeLX	CACTTGACTTCAAGACCAAATCTAACC	AATTGCCTTTTTAATGCTTTCTACACCAT	ATGCAGGGATTAAACC	Solyc05g007940
LeTBN1	GAGGCAGCTCATGCAGTCA	GGCCGATAAGTCACCGTTAACATA	TCCGGCAACAACATCT	Solyc02g078910
LeRBOH1	AGTTTCGTCAGTTTTCACAGGAGTT	CTTTAAGCTCTTGCGAGAAATGCT	AACAGCCTCAGCTTTC	Solyc08g081690
LeTAPG1	ACGGATGTCATAACGTAAAGCTTCA	CATGAATTCCATCTGTGTTGGGACTA	CTGGAGCGGATACCTT	Solyc02g067630
LeTAPG4	CCCAGGCCATGGAATTAGCATT	ACTGTCACATTTTGTACTCCTTGCT	CCCAACCTAAGCTTCC	Solyc12g096750
