# Supplementary material for: 1-Aminocyclopropane-1-Carboxylate Oxidase Induction in Tomato Flower Pedicel Phloem and Abscission Related Processes Are Differentially Sensitive to Ethylene
Source: Front Plant Sci. 2017 Mar 31;8:464. doi: 10.3389/fpls.2017.00464 (PMC5374216; doi:10.3389/fpls.2017.00464)
Supplement: Supplementary file 8 [file Table2.PDF]

1-aminocyclopropane-1-carboxylate oxidase induction in tomato flower pedicel phloem and abscission related processes are differentially sensitive to ethylene

Marko Chersicola, Aleš Kladnik, Magda Tušek Žnidarič, Tanja Mrak, Kristina Gruden, Marina Dermastia.

Correspondence: marina.dermastia@nib.si

Supplementary Table S2.A: Two-way ANOVA of time series expression data for the selected genes. Two-way ANOVA was used to examined the effects of time after induction (0, 2, 4, 6, 8, 10, 12 h) and abscission zone side (proximal vs. distal) on the expression of the selected genes. Significance levels of the main effects of the timepoint and zone factors on gene expression are given, and for the interaction effects between timepoint and zone. Statistical significance was set at $p < 0.05$.

**Gene**	***p*-value**		
	**Factor 1 (timepoint)**	**Factor 2 (zone)**	**Interaction between factors**
LeACO1	<0.001	0.292	0.958
LeACO4	<0.001	0.032	0.543
LeEIL2	0.011	0.860	0.901
LeLX	<0.001	0.002	0.223
LeTBN1	<0.001	<0.001	0.066
LeRBOH1	<0.001	0.581	0.230
LeTAPG1	<0.001	0.946	0.492
LeTAPG4	<0.001	0.533	0.947

Supplemental table 2.1.A: Post-hoc analysis results for *LeACO1* gene expression data. Post-hoc analysis results for gene expression data using the Tukey's Honest Significant Difference method. Results are displayed as a table with columns "Comparison" giving each combination of compared factors pairs (P for proximal, D for distal and 0-12 for timepoints), "Difference" giving the difference in the observed means, "Lower" giving the lower end point of the interval, "Upper" giving the upper end point and "Adjusted *p*-value" giving the *p*-value after adjustment for the multiple comparisons.

Comparison	Difference	Lower	Upper	Adjusted p-value
P:0-D:0	-0.71018990	-2.6872379	1.2668581	0.9843326
D:2-D:0	3.61450195	1.6374539	5.5915500	0.0000221
P:2-D:0	3.22537474	1.2483267	5.2024228	0.0001441
D:4-D:0	4.27921159	2.3021636	6.2562596	0.0000010
P:4-D:0	4.00710225	2.0300542	5.9841503	0.0000035
D:6-D:0	3.95073866	1.9736906	5.9277867	0.0000045
P:6-D:0	3.57387285	1.5968248	5.5509209	0.0000269
D:8-D:0	4.13618765	2.1591396	6.1132357	0.0000019
P:8-D:0	3.67247562	1.6954276	5.6495237	0.0000168
D:10-D:0	4.29948500	2.3224370	6.2765330	0.0000009
P:10-D:0	3.79872078	1.8216727	5.7757688	0.0000092
D:12-D:0	3.54287668	1.5658286	5.5199247	0.0000312
P:12-D:0	3.07722696	1.1001789	5.0542750	0.0002956
D:2-P:0	4.32469185	2.3476438	6.3017399	0.0000008
P:2-P:0	3.93556464	1.9585166	5.9126127	0.0000049
D:4-P:0	4.98940149	3.0123535	6.9664495	0.0000000
P:4-P:0	4.71729215	2.7402441	6.6943402	0.0000001
D:6-P:0	4.66092856	2.6838805	6.6379766	0.0000002
P:6-P:0	4.28406274	2.3070147	6.2611108	0.0000010
D:8-P:0	4.84637755	2.8693295	6.8234256	0.0000001
P:8-P:0	4.38266552	2.4056175	6.3597136	0.0000006
D:10-P:0	5.00967490	3.0326269	6.9867229	0.0000000
P:10-P:0	4.50891068	2.5318626	6.4859587	0.0000004
D:12-P:0	4.25306657	2.2760185	6.2301146	0.0000011
P:12-P:0	3.78741686	1.8103688	5.7644649	0.0000097
P:2-D:2	-0.38912721	-2.3661753	1.5879208	0.9999612
D:4-D:2	0.66470964	-1.3123384	2.6417577	0.9910637
P:4-D:2	0.39260030	-1.5844477	2.3696483	0.9999571
D:6-D:2	0.33623671	-1.6408113	2.3132847	0.9999928
P:6-D:2	-0.04062911	-2.0176771	1.9364189	1.0000000
D:8-D:2	0.52168570	-1.4553623	2.4987337	0.9990945
P:8-D:2	0.05797367	-1.9190744	2.0350217	1.0000000
D:10-D:2	0.68498305	-1.2920650	2.6620311	0.9884259
P:10-D:2	0.18421882	-1.7928292	2.1612669	1.0000000
D:12-D:2	-0.07162528	-2.0486733	1.9054228	1.0000000
P:12-D:2	-0.53727499	-2.5143230	1.4397730	0.9987830
D:4-P:2	1.05383685	-0.9232112	3.0308849	0.7827467
P:4-P:2	0.78172751	-1.1953205	2.7587756	0.9665759
D:6-P:2	0.72536392	-1.2516841	2.7024120	0.9813770
P:6-P:2	0.34849810	-1.6285499	2.3255461	0.9999890

D:8-P:2	0.91081291	-1.0662351	2.8878609	0.9040220
P:8-P:2	0.44710088	-1.5299472	2.4241489	0.9998199
D:10-P:2	1.07411026	-0.9029378	3.0511583	0.7617119
P:10-P:2	0.57334604	-1.4037020	2.5503941	0.9976999
D:12-P:2	0.31750193	-1.6595461	2.2945500	0.9999963
P:12-P:2	-0.14814778	-2.1251958	1.8289003	1.0000000
P:4-D:4	-0.27210934	-2.2491574	1.7049387	0.9999994
D:6-D:4	-0.32847293	-2.3055210	1.6485751	0.9999945
P:6-D:4	-0.70533875	-2.6823868	1.2717093	0.9851965
D:8-D:4	-0.14302394	-2.1200720	1.8340241	1.0000000
P:8-D:4	-0.60673597	-2.5837840	1.3703121	0.9960715
D:10-D:4	0.02027341	-1.9567746	1.9973215	1.0000000
P:10-D:4	-0.48049081	-2.4575389	1.4965572	0.9996123
D:12-D:4	-0.73633492	-2.7133830	1.2407131	0.9789874
P:12-D:4	-1.20198463	-3.1790327	0.7750634	0.6158983
D:6-P:4	-0.05636359	-2.0334116	1.9206844	1.0000000
P:6-P:4	-0.43322941	-2.4102774	1.5438186	0.9998721
D:8-P:4	0.12908540	-1.8479626	2.1061334	1.0000000
P:8-P:4	-0.33462663	-2.3116747	1.6424214	0.9999932
D:10-P:4	0.29238275	-1.6846653	2.2694308	0.9999986
P:10-P:4	-0.20838148	-2.1854295	1.7686666	1.0000000
D:12-P:4	-0.46422558	-2.4412736	1.5128225	0.9997306
P:12-P:4	-0.92987529	-2.9069233	1.0471727	0.8909548
P:6-D:6	-0.37686581	-2.3539139	1.6001822	0.9999730
D:8-D:6	0.18544899	-1.7915990	2.1624970	1.0000000
P:8-D:6	-0.27826303	-2.2553111	1.6987850	0.9999992
D:10-D:6	0.34874634	-1.6283017	2.3257944	0.9999889
P:10-D:6	-0.15201788	-2.1290659	1.8250302	1.0000000
D:12-D:6	-0.40786198	-2.3849100	1.5691861	0.9999342
P:12-D:6	-0.87351170	-2.8505597	1.1035363	0.9266657
D:8-P:6	0.56231480	-1.4147332	2.5393628	0.9980940
P:8-P:6	0.09860278	-1.8784453	2.0756508	1.0000000
D:10-P:6	0.72561216	-1.2514359	2.7026602	0.9813253
P:10-P:6	0.22484793	-1.7522001	2.2018960	0.9999999
D:12-P:6	-0.03099617	-2.0080442	1.9460519	1.0000000
P:12-P:6	-0.49664588	-2.4736939	1.4804022	0.9994527
P:8-D:8	-0.46371203	-2.4407601	1.5133360	0.9997337
D:10-D:8	0.16329735	-1.8137507	2.1403454	1.0000000
P:10-D:8	-0.33746687	-2.3145149	1.6395812	0.9999924
D:12-D:8	-0.59331097	-2.5703590	1.3837371	0.9968136
P:12-D:8	-1.05896069	-3.0360087	0.9180873	0.7775049
D:10-P:8	0.62700938	-1.3500387	2.6040574	0.9946860
P:10-P:8	0.12624515	-1.8508029	2.1032932	1.0000000
D:12-P:8	-0.12959895	-2.1066470	1.8474491	1.0000000
P:12-P:8	-0.59524866	-2.5722967	1.3817994	0.9967143
P:10-D:10	-0.50076423	-2.4778123	1.4762838	0.9994039
D:12-D:10	-0.75660833	-2.7336564	1.2204397	0.9739698
P:12-D:10	-1.22225804	-3.1993061	0.7547900	0.5916924
D:12-P:10	-0.25584410	-2.2328921	1.7212039	0.9999997
P:12-P:10	-0.72149382	-2.6985419	1.2555542	0.9821683
P:12-D:12	-0.46564971	-2.4426978	1.5113983	0.9997217

Supplemental table 2.2.A: Post-hoc analysis results for *LeACO4* gene expression data. Post-hoc analysis results for gene expression data using the Tukey's Honest Significant Difference method. Results are displayed as a table with columns "Comparison" giving each combination of compared factors pairs (P for proximal, D for distal and 0-12 for timepoints), "Difference" giving the difference in the observed means, "Lower" giving the lower end point of the interval, "Upper" giving the upper end point and "Adjusted *p*-value" giving the *p*-value after adjustment for the multiple comparisons.

Comparison	Difference	Lower	Upper	Adjusted p-value
P:0-D:0	-0.71018990	-2.6872379	1.2668581	0.9843326
D:2-D:0	3.61450195	1.6374539	5.5915500	0.0000221
P:2-D:0	3.22537474	1.2483267	5.2024228	0.0001441
D:4-D:0	4.27921159	2.3021636	6.2562596	0.0000010
P:4-D:0	4.00710225	2.0300542	5.9841503	0.0000035
D:6-D:0	3.95073866	1.9736906	5.9277867	0.0000045
P:6-D:0	3.57387285	1.5968248	5.5509209	0.0000269
D:8-D:0	4.13618765	2.1591396	6.1132357	0.0000019
P:8-D:0	3.67247562	1.6954276	5.6495237	0.0000168
D:10-D:0	4.29948500	2.3224370	6.2765330	0.0000009
P:10-D:0	3.79872078	1.8216727	5.7757688	0.0000092
D:12-D:0	3.54287668	1.5658286	5.5199247	0.0000312
P:12-D:0	3.07722696	1.1001789	5.0542750	0.0002956
D:2-P:0	4.32469185	2.3476438	6.3017399	0.0000008
P:2-P:0	3.93556464	1.9585166	5.9126127	0.0000049
D:4-P:0	4.98940149	3.0123535	6.9664495	0.0000000
P:4-P:0	4.71729215	2.7402441	6.6943402	0.0000001
D:6-P:0	4.66092856	2.6838805	6.6379766	0.0000002
P:6-P:0	4.28406274	2.3070147	6.2611108	0.0000010
D:8-P:0	4.84637755	2.8693295	6.8234256	0.0000001
P:8-P:0	4.38266552	2.4056175	6.3597136	0.0000006
D:10-P:0	5.00967490	3.0326269	6.9867229	0.0000000
P:10-P:0	4.50891068	2.5318626	6.4859587	0.0000004
D:12-P:0	4.25306657	2.2760185	6.2301146	0.0000011
P:12-P:0	3.78741686	1.8103688	5.7644649	0.0000097
P:2-D:2	-0.38912721	-2.3661753	1.5879208	0.9999612
D:4-D:2	0.66470964	-1.3123384	2.6417577	0.9910637
P:4-D:2	0.39260030	-1.5844477	2.3696483	0.9999571
D:6-D:2	0.33623671	-1.6408113	2.3132847	0.9999928
P:6-D:2	-0.04062911	-2.0176771	1.9364189	1.0000000
D:8-D:2	0.52168570	-1.4553623	2.4987337	0.9990945
P:8-D:2	0.05797367	-1.9190744	2.0350217	1.0000000
D:10-D:2	0.68498305	-1.2920650	2.6620311	0.9884259
P:10-D:2	0.18421882	-1.7928292	2.1612669	1.0000000
D:12-D:2	-0.07162528	-2.0486733	1.9054228	1.0000000
P:12-D:2	-0.53727499	-2.5143230	1.4397730	0.9987830
D:4-P:2	1.05383685	-0.9232112	3.0308849	0.7827467
P:4-P:2	0.78172751	-1.1953205	2.7587756	0.9665759
D:6-P:2	0.72536392	-1.2516841	2.7024120	0.9813770
P:6-P:2	0.34849810	-1.6285499	2.3255461	0.9999890
D:8-P:2	0.91081291	-1.0662351	2.8878609	0.9040220

P:8-P:2	0.44710088	-1.5299472	2.4241489	0.9998199
D:10-P:2	1.07411026	-0.9029378	3.0511583	0.7617119
P:10-P:2	0.57334604	-1.4037020	2.5503941	0.9976999
D:12-P:2	0.31750193	-1.6595461	2.2945500	0.9999963
P:12-P:2	-0.14814778	-2.1251958	1.8289003	1.0000000
P:4-D:4	-0.27210934	-2.2491574	1.7049387	0.9999994
D:6-D:4	-0.32847293	-2.3055210	1.6485751	0.9999945
P:6-D:4	-0.70533875	-2.6823868	1.2717093	0.9851965
D:8-D:4	-0.14302394	-2.1200720	1.8340241	1.0000000
P:8-D:4	-0.60673597	-2.5837840	1.3703121	0.9960715
D:10-D:4	0.02027341	-1.9567746	1.9973215	1.0000000
P:10-D:4	-0.48049081	-2.4575389	1.4965572	0.9996123
D:12-D:4	-0.73633492	-2.7133830	1.2407131	0.9789874
P:12-D:4	-1.20198463	-3.1790327	0.7750634	0.6158983
D:6-P:4	-0.05636359	-2.0334116	1.9206844	1.0000000
P:6-P:4	-0.43322941	-2.4102774	1.5438186	0.9998721
D:8-P:4	0.12908540	-1.8479626	2.1061334	1.0000000
P:8-P:4	-0.33462663	-2.3116747	1.6424214	0.9999932
D:10-P:4	0.29238275	-1.6846653	2.2694308	0.9999986
P:10-P:4	-0.20838148	-2.1854295	1.7686666	1.0000000
D:12-P:4	-0.46422558	-2.4412736	1.5128225	0.9997306
P:12-P:4	-0.92987529	-2.9069233	1.0471727	0.8909548
P:6-D:6	-0.37686581	-2.3539139	1.6001822	0.9999730
D:8-D:6	0.18544899	-1.7915990	2.1624970	1.0000000
P:8-D:6	-0.27826303	-2.2553111	1.6987850	0.9999992
D:10-D:6	0.34874634	-1.6283017	2.3257944	0.9999889
P:10-D:6	-0.15201788	-2.1290659	1.8250302	1.0000000
D:12-D:6	-0.40786198	-2.3849100	1.5691861	0.9999342
P:12-D:6	-0.87351170	-2.8505597	1.1035363	0.9266657
D:8-P:6	0.56231480	-1.4147332	2.5393628	0.9980940
P:8-P:6	0.09860278	-1.8784453	2.0756508	1.0000000
D:10-P:6	0.72561216	-1.2514359	2.7026602	0.9813253
P:10-P:6	0.22484793	-1.7522001	2.2018960	0.9999999
D:12-P:6	-0.03099617	-2.0080442	1.9460519	1.0000000
P:12-P:6	-0.49664588	-2.4736939	1.4804022	0.9994527
P:8-D:8	-0.46371203	-2.4407601	1.5133360	0.9997337
D:10-D:8	0.16329735	-1.8137507	2.1403454	1.0000000
P:10-D:8	-0.33746687	-2.3145149	1.6395812	0.9999924
D:12-D:8	-0.59331097	-2.5703590	1.3837371	0.9968136
P:12-D:8	-1.05896069	-3.0360087	0.9180873	0.7775049
D:10-P:8	0.62700938	-1.3500387	2.6040574	0.9946860
P:10-P:8	0.12624515	-1.8508029	2.1032932	1.0000000
D:12-P:8	-0.12959895	-2.1066470	1.8474491	1.0000000
P:12-P:8	-0.59524866	-2.5722967	1.3817994	0.9967143
P:10-D:10	-0.50076423	-2.4778123	1.4762838	0.9994039
D:12-D:10	-0.75660833	-2.7336564	1.2204397	0.9739698
P:12-D:10	-1.22225804	-3.1993061	0.7547900	0.5916924
D:12-P:10	-0.25584410	-2.2328921	1.7212039	0.9999997
P:12-P:10	-0.72149382	-2.6985419	1.2555542	0.9821683
P:12-D:12	-0.46564971	-2.4426978	1.5113983	0.9997217

Supplemental table 2.3.A: Post-hoc analysis results for *LeEIL2*ene expression data. Post-hoc analysis results for gene expression data using the Tukey's Honest Significant Difference method. Results are displayed as a table with columns "Comparison" giving each combination of compared factors pairs (P for proximal, D for distal and 0-12 for timepoints), "Difference" giving the difference in the observed means, "Lower" giving the lower end point of the interval, "Upper" giving the upper end point and "Adjusted *p*-value" giving the *p*-value after adjustment for the multiple comparisons.

Comparison	Difference	Lower	Upper	Adjusted p-value
P:0-D:0	-0.71018990	-2.6872379	1.2668581	0.9843326
D:2-D:0	3.61450195	1.6374539	5.5915500	0.0000221
P:2-D:0	3.22537474	1.2483267	5.2024228	0.0001441
D:4-D:0	4.27921159	2.3021636	6.2562596	0.0000010
P:4-D:0	4.00710225	2.0300542	5.9841503	0.0000035
D:6-D:0	3.95073866	1.9736906	5.9277867	0.0000045
P:6-D:0	3.57387285	1.5968248	5.5509209	0.0000269
D:8-D:0	4.13618765	2.1591396	6.1132357	0.0000019
P:8-D:0	3.67247562	1.6954276	5.6495237	0.0000168
D:10-D:0	4.29948500	2.3224370	6.2765330	0.0000009
P:10-D:0	3.79872078	1.8216727	5.7757688	0.0000092
D:12-D:0	3.54287668	1.5658286	5.5199247	0.0000312
P:12-D:0	3.07722696	1.1001789	5.0542750	0.0002956
D:2-P:0	4.32469185	2.3476438	6.3017399	0.0000008
P:2-P:0	3.93556464	1.9585166	5.9126127	0.0000049
D:4-P:0	4.98940149	3.0123535	6.9664495	0.0000000
P:4-P:0	4.71729215	2.7402441	6.6943402	0.0000001
D:6-P:0	4.66092856	2.6838805	6.6379766	0.0000002
P:6-P:0	4.28406274	2.3070147	6.2611108	0.0000010
D:8-P:0	4.84637755	2.8693295	6.8234256	0.0000001
P:8-P:0	4.38266552	2.4056175	6.3597136	0.0000006
D:10-P:0	5.00967490	3.0326269	6.9867229	0.0000000
P:10-P:0	4.50891068	2.5318626	6.4859587	0.0000004
D:12-P:0	4.25306657	2.2760185	6.2301146	0.0000011
P:12-P:0	3.78741686	1.8103688	5.7644649	0.0000097
P:2-D:2	-0.38912721	-2.3661753	1.5879208	0.9999612
D:4-D:2	0.66470964	-1.3123384	2.6417577	0.9910637
P:4-D:2	0.39260030	-1.5844477	2.3696483	0.9999571
D:6-D:2	0.33623671	-1.6408113	2.3132847	0.9999928
P:6-D:2	-0.04062911	-2.0176771	1.9364189	1.0000000
D:8-D:2	0.52168570	-1.4553623	2.4987337	0.9990945
P:8-D:2	0.05797367	-1.9190744	2.0350217	1.0000000
D:10-D:2	0.68498305	-1.2920650	2.6620311	0.9884259
P:10-D:2	0.18421882	-1.7928292	2.1612669	1.0000000

D:12-D:2	-0.07162528	-2.0486733	1.9054228	1.0000000
P:12-D:2	-0.53727499	-2.5143230	1.4397730	0.9987830
D:4-P:2	1.05383685	-0.9232112	3.0308849	0.7827467
P:4-P:2	0.78172751	-1.1953205	2.7587756	0.9665759
D:6-P:2	0.72536392	-1.2516841	2.7024120	0.9813770
P:6-P:2	0.34849810	-1.6285499	2.3255461	0.9999890
D:8-P:2	0.91081291	-1.0662351	2.8878609	0.9040220
P:8-P:2	0.44710088	-1.5299472	2.4241489	0.9998199
D:10-P:2	1.07411026	-0.9029378	3.0511583	0.7617119
P:10-P:2	0.57334604	-1.4037020	2.5503941	0.9976999
D:12-P:2	0.31750193	-1.6595461	2.2945500	0.9999963
P:12-P:2	-0.14814778	-2.1251958	1.8289003	1.0000000
P:4-D:4	-0.27210934	-2.2491574	1.7049387	0.9999994
D:6-D:4	-0.32847293	-2.3055210	1.6485751	0.9999945
P:6-D:4	-0.70533875	-2.6823868	1.2717093	0.9851965
D:8-D:4	-0.14302394	-2.1200720	1.8340241	1.0000000
P:8-D:4	-0.60673597	-2.5837840	1.3703121	0.9960715
D:10-D:4	0.02027341	-1.9567746	1.9973215	1.0000000
P:10-D:4	-0.48049081	-2.4575389	1.4965572	0.9996123
D:12-D:4	-0.73633492	-2.7133830	1.2407131	0.9789874
P:12-D:4	-1.20198463	-3.1790327	0.7750634	0.6158983
D:6-P:4	-0.05636359	-2.0334116	1.9206844	1.0000000
P:6-P:4	-0.43322941	-2.4102774	1.5438186	0.9998721
D:8-P:4	0.12908540	-1.8479626	2.1061334	1.0000000
P:8-P:4	-0.33462663	-2.3116747	1.6424214	0.9999932
D:10-P:4	0.29238275	-1.6846653	2.2694308	0.9999986
P:10-P:4	-0.20838148	-2.1854295	1.7686666	1.0000000
D:12-P:4	-0.46422558	-2.4412736	1.5128225	0.9997306
P:12-P:4	-0.92987529	-2.9069233	1.0471727	0.8909548
P:6-D:6	-0.37686581	-2.3539139	1.6001822	0.9999730
D:8-D:6	0.18544899	-1.7915990	2.1624970	1.0000000
P:8-D:6	-0.27826303	-2.2553111	1.6987850	0.9999992
D:10-D:6	0.34874634	-1.6283017	2.3257944	0.9999889
P:10-D:6	-0.15201788	-2.1290659	1.8250302	1.0000000
D:12-D:6	-0.40786198	-2.3849100	1.5691861	0.9999342
P:12-D:6	-0.87351170	-2.8505597	1.1035363	0.9266657
D:8-P:6	0.56231480	-1.4147332	2.5393628	0.9980940
P:8-P:6	0.09860278	-1.8784453	2.0756508	1.0000000
D:10-P:6	0.72561216	-1.2514359	2.7026602	0.9813253
P:10-P:6	0.22484793	-1.7522001	2.2018960	0.9999999
D:12-P:6	-0.03099617	-2.0080442	1.9460519	1.0000000
P:12-P:6	-0.49664588	-2.4736939	1.4804022	0.9994527
P:8-D:8	-0.46371203	-2.4407601	1.5133360	0.9997337
D:10-D:8	0.16329735	-1.8137507	2.1403454	1.0000000
P:10-D:8	-0.33746687	-2.3145149	1.6395812	0.9999924
D:12-D:8	-0.59331097	-2.5703590	1.3837371	0.9968136
P:12-D:8	-1.05896069	-3.0360087	0.9180873	0.7775049
D:10-P:8	0.62700938	-1.3500387	2.6040574	0.9946860
P:10-P:8	0.12624515	-1.8508029	2.1032932	1.0000000
D:12-P:8	-0.12959895	-2.1066470	1.8474491	1.0000000
P:12-P:8	-0.59524866	-2.5722967	1.3817994	0.9967143
P:10-D:10	-0.50076423	-2.4778123	1.4762838	0.9994039
D:12-D:10	-0.75660833	-2.7336564	1.2204397	0.9739698
P:12-D:10	-1.22225804	-3.1993061	0.7547900	0.5916924
D:12-P:10	-0.25584410	-2.2328921	1.7212039	0.9999997
P:12-P:10	-0.72149382	-2.6985419	1.2555542	0.9821683
P:12-D:12	-0.46564971	-2.4426978	1.5113983	0.9997217

Supplemental table 2.4.A: Post-hoc analysis results for *LeLX* gene expression data. Post-hoc analysis results for gene expression data using the Tukey's Honest Significant Difference method. Results are displayed as a table with columns "Comparison" giving each combination of compared factors pairs (P for proximal, D for distal and 0-12 for timepoints), "Difference" giving the difference in the observed means, "Lower" giving the lower end point of the interval, "Upper" giving the upper end point and "Adjusted *p*-value" giving the *p*-value after adjustment for the multiple comparisons.

Comparison	Difference	Lower	Upper	Adjusted p-value
P:0-D:0	-0.08438116	-1.84463820	1.6758759	1.0000000
D:2-D:0	1.82633929	0.06608224	3.5865963	0.0364943
P:2-D:0	0.80586199	-0.95439506	2.5661190	0.9077341
D:4-D:0	4.98523641	3.22497936	6.7454935	0.0000000
P:4-D:0	4.04506884	2.28481179	5.8053259	0.0000003
D:6-D:0	4.55675996	2.79650291	6.3170170	0.0000000
P:6-D:0	3.10158287	1.34132582	4.8618399	0.0000414
D:8-D:0	6.80349242	5.04323537	8.5637495	0.0000000
P:8-D:0	5.58863034	3.82837329	7.3488874	0.0000000
D:10-D:0	8.25162229	6.49136524	10.0118793	0.0000000
P:10-D:0	6.95763509	5.19737804	8.7178921	0.0000000
D:12-D:0	7.84914383	6.08888678	9.6094009	0.0000000
P:12-D:0	6.19253538	4.43227833	7.9527924	0.0000000
D:2-P:0	1.91072044	0.15046339	3.6709775	0.0241422
P:2-P:0	0.89024314	-0.87001391	2.6505002	0.8345116
D:4-P:0	5.06961756	3.30936051	6.8298746	0.0000000
P:4-P:0	4.12944999	2.36919294	5.8897070	0.0000002
D:6-P:0	4.64114111	2.88088406	6.4013982	0.0000000
P:6-P:0	3.18596403	1.42570698	4.9462211	0.0000263
D:8-P:0	6.88787357	5.12761652	8.6481306	0.0000000
P:8-P:0	5.67301150	3.91275445	7.4332685	0.0000000
D:10-P:0	8.33600345	6.57574640	10.0962605	0.0000000
P:10-P:0	7.04201625	5.28175920	8.8022733	0.0000000
D:12-P:0	7.93352498	6.17326793	9.6937820	0.0000000
P:12-P:0	6.27691653	4.51665948	8.0371736	0.0000000
P:2-D:2	-1.02047730	-2.78073435	0.7397797	0.6816257
D:4-D:2	3.15889712	1.39864007	4.9191542	0.0000304
P:4-D:2	2.21872955	0.45847250	3.9789866	0.0049424
D:6-D:2	2.73042067	0.97016362	4.4906777	0.0003111
P:6-D:2	1.27524358	-0.48501347	3.0355006	0.3545339
D:8-D:2	4.97715313	3.21689608	6.7374102	0.0000000
P:8-D:2	3.76229105	2.00203400	5.5225481	0.0000013
D:10-D:2	6.42528300	4.66502595	8.1855401	0.0000000
P:10-D:2	5.13129581	3.37103876	6.8915529	0.0000000
D:12-D:2	6.02280454	4.26254749	7.7830616	0.0000000
P:12-D:2	4.36619609	2.60593904	6.1264531	0.0000001
D:4-P:2	4.17937442	2.41911737	5.9396315	0.0000002
P:4-P:2	3.23920685	1.47894980	4.9994639	0.0000198

D:6-P:2	3.75089797	1.99064092	5.5111550	0.0000014
P:6-P:2	2.29572088	0.53546383	4.0559779	0.0032811
D:8-P:2	5.99763043	4.23737338	7.7578875	0.0000000
P:8-P:2	4.78276835	3.02251130	6.5430254	0.0000000
D:10-P:2	7.44576030	5.68550325	9.2060174	0.0000000
P:10-P:2	6.15177311	4.39151606	7.9120302	0.0000000
D:12-P:2	7.04328184	5.28302479	8.8035389	0.0000000
P:12-P:2	5.38667339	3.62641634	7.1469304	0.0000000
P:4-D:4	-0.94016757	-2.70042462	0.8200895	0.7805837
D:6-D:4	-0.42847645	-2.18873350	1.3317806	0.9996058
P:6-D:4	-1.88365353	-3.64391058	-0.1233965	0.0275983
D:8-D:4	1.81825601	0.05799896	3.5785131	0.0379439
P:8-D:4	0.60339393	-1.15686312	2.3636510	0.9894350
D:10-D:4	3.26638589	1.50612884	5.0266429	0.0000171
P:10-D:4	1.97239869	0.21214164	3.7326557	0.0177269
D:12-D:4	2.86390742	1.10365037	4.6241645	0.0001503
P:12-D:4	1.20729897	-0.55295808	2.9675560	0.4354959
D:6-P:4	0.51169112	-1.24856593	2.2719482	0.9976468
P:6-P:4	-0.94348596	-2.70374301	0.8167711	0.7767603
D:8-P:4	2.75842358	0.99816653	4.5186806	0.0002670
P:8-P:4	1.54356150	-0.21669555	3.3038186	0.1311608
D:10-P:4	4.20655345	2.44629640	5.9668105	0.0000001
P:10-P:4	2.91256626	1.15230921	4.6728233	0.0001153
D:12-P:4	3.80407499	2.04381794	5.5643320	0.0000010
P:12-P:4	2.14746654	0.38720949	3.9077236	0.0071943
P:6-D:6	-1.45517709	-3.21543413	0.3050800	0.1872556
D:8-D:6	2.24673246	0.48647541	4.0069895	0.0042601
P:8-D:6	1.03187038	-0.72838667	2.7921274	0.6667499
D:10-D:6	3.69486233	1.93460528	5.4551194	0.0000018
P:10-D:6	2.40087514	0.64061809	4.1611322	0.0018651
D:12-D:6	3.29238387	1.53212682	5.0526409	0.0000149
P:12-D:6	1.63577542	-0.12448163	3.3960325	0.0882615
D:8-P:6	3.70190955	1.94165250	5.4621666	0.0000017
P:8-P:6	2.48704747	0.72679042	4.2473045	0.0011701
D:10-P:6	5.15003942	3.38978237	6.9102965	0.0000000
P:10-P:6	3.85605222	2.09579517	5.6163093	0.0000008
D:12-P:6	4.74756095	2.98730390	6.5078180	0.0000000
P:12-P:6	3.09095250	1.33069545	4.8512096	0.0000439
P:8-D:8	-1.21486208	-2.97511913	0.5453950	0.4260894
D:10-D:8	1.44812987	-0.31212718	3.2083869	0.1924358
P:10-D:8	0.15414268	-1.60611437	1.9143997	1.0000000
D:12-D:8	1.04565141	-0.71460564	2.8059085	0.6485811
P:12-D:8	-0.61095704	-2.37121409	1.1493000	0.9882497
D:10-P:8	2.66299195	0.90273490	4.4232490	0.0004493
P:10-P:8	1.36900475	-0.39125230	3.1292618	0.2582518
D:12-P:8	2.26051348	0.50025643	4.0207705	0.0039590
P:12-P:8	0.60390504	-1.15635201	2.3641621	0.9893580
P:10-D:10	-1.29398720	-3.05424425	0.4662699	0.3337564
D:12-D:10	-0.40247847	-2.16273552	1.3577786	0.9997973
P:12-D:10	-2.05908691	-3.81934396	-0.2988299	0.0113893
D:12-P:10	0.89150873	-0.86874832	2.6517658	0.8332342
P:12-P:10	-0.76509972	-2.52535677	0.9951573	0.9342676
P:12-D:12	-1.65660845	-3.41686550	0.1036486	0.0804555

Supplemental table 2.5.A: Post-hoc analysis results for *LeTBN1* gene expression data. Post-hoc analysis results for gene expression data using the Tukey's Honest Significant Difference method. Results are displayed as a table with columns "Comparison" giving each combination of compared factors pairs (P for proximal, D for distal and 0-12 for timepoints), "Difference" giving the difference in the observed means, "Lower" giving the lower end point of the interval, "Upper" giving the upper end point and "Adjusted *p*-value" giving the *p*-value after adjustment for the multiple comparisons.

Comparison	Difference	Lower	Upper	Adjusted p-value
P:0-D:0	-0.3734259	-2.422075858	1.67522406	0.9999838
D:2-D:0	0.3149854	-1.733664534	2.36363538	0.9999978
P:2-D:0	-0.5755114	-2.624161391	1.47313852	0.9983109
D:4-D:0	3.7354286	1.686778647	5.78407856	0.0000232
P:4-D:0	1.4754433	-0.573206680	3.52409323	0.3630508
D:6-D:0	4.7397429	2.691092944	6.78839286	0.0000003
P:6-D:0	2.6729343	0.624284312	4.72158423	0.0032646
D:8-D:0	5.5036580	3.455008009	7.55230792	0.0000000
P:8-D:0	3.3994445	1.350794575	5.44809449	0.0001102
D:10-D:0	6.1603661	4.111716125	8.20901604	0.0000000
P:10-D:0	4.2886258	2.239975819	6.33727573	0.0000019
D:12-D:0	6.3565677	4.307917716	8.40521763	0.0000000
P:12-D:0	4.0210704	1.972420414	6.06972033	0.0000063
D:2-P:0	0.6884113	-1.360238632	2.73706128	0.9911056
P:2-P:0	-0.2020855	-2.250735489	1.84656442	1.0000000
D:4-P:0	4.1088545	2.060204548	6.15750446	0.0000042
P:4-P:0	1.8488692	-0.199780779	3.89751914	0.1084044
D:6-P:0	5.1131688	3.064518846	7.16181876	0.0000001
P:6-P:0	3.0463602	0.997710213	5.09501013	0.0005756
D:8-P:0	5.8770839	3.828433910	7.92573382	0.0000000
P:8-P:0	3.7728704	1.724220476	5.82152039	0.0000195
D:10-P:0	6.5337920	4.485142026	8.58244194	0.0000000
P:10-P:0	4.6620517	2.613401721	6.71070163	0.0000004
D:12-P:0	6.7299936	4.681343617	8.77864353	0.0000000
P:12-P:0	4.3944963	2.345846315	6.44314623	0.0000012
P:2-D:2	-0.8904969	-2.939146814	1.15815310	0.9342444
D:4-D:2	3.4204432	1.371793224	5.46909314	0.0000999
P:4-D:2	1.1604579	-0.888192103	3.20910781	0.7116289
D:6-D:2	4.4247575	2.376107521	6.47340743	0.0000010
P:6-D:2	2.3579488	0.309298888	4.40659880	0.0134975
D:8-D:2	5.1886725	3.140022585	7.23732250	0.0000000
P:8-D:2	3.0844591	1.035809152	5.13310907	0.0004816
D:10-D:2	5.8453807	3.796730701	7.89403062	0.0000000
P:10-D:2	3.9736404	1.924990396	6.02229031	0.0000078
D:12-D:2	6.0415822	3.992932292	8.09023221	0.0000000
P:12-D:2	3.7060849	1.657434990	5.75473490	0.0000265
D:4-P:2	4.3109400	2.262290081	6.35958999	0.0000017
P:4-P:2	2.0509547	0.002304754	4.09960467	0.0495365
D:6-P:2	5.3152543	3.266604378	7.36390429	0.0000000
P:6-P:2	3.2484457	1.199795745	5.29709566	0.0002234

D:8-P:2	6.0791694	4.030519442	8.12781936	0.0000000
P:8-P:2	3.9749560	1.926306009	6.02360592	0.0000078
D:10-P:2	6.7358775	4.687227558	8.78452747	0.0000000
P:10-P:2	4.8641372	2.815487253	6.91278717	0.0000002
D:12-P:2	6.9320791	4.883429149	8.98072906	0.0000000
P:12-P:2	4.5965818	2.547931848	6.64523176	0.0000005
P:4-D:4	-2.2599853	-4.308635284	-0.21133537	0.0206719
D:6-D:4	1.0043143	-1.044335660	3.05296425	0.8608339
P:6-D:4	-1.0624943	-3.111144293	0.98615562	0.8108862
D:8-D:4	1.7682294	-0.280420595	3.81687932	0.1449752
P:8-D:4	-0.3359841	-2.384634029	1.71266588	0.9999953
D:10-D:4	2.4249375	0.376287520	4.47358743	0.0100358
P:10-D:4	0.5531972	-1.495452785	2.60184713	0.9988580
D:12-D:4	2.6211391	0.572489111	4.66978903	0.0041390
P:12-D:4	0.2856418	-1.763008190	2.33429172	0.9999993
D:6-P:4	3.2642996	1.215649667	5.31294958	0.0002074
P:6-P:4	1.1974910	-0.851158966	3.24614095	0.6706191
D:8-P:4	4.0282147	1.979564731	6.07686465	0.0000061
P:8-P:4	1.9240013	-0.124648702	3.97265121	0.0817060
D:10-P:4	4.6849228	2.636272847	6.73357276	0.0000003
P:10-P:4	2.8131825	0.764532542	4.86183246	0.0017081
D:12-P:4	4.8811244	2.832474438	6.92977435	0.0000001
P:12-P:4	2.5456271	0.496977137	4.59427705	0.0058361
P:6-D:6	-2.0668086	-4.115458590	-0.01815868	0.0464527
D:8-D:6	0.7639151	-1.284734893	2.81256502	0.9787860
P:8-D:6	-1.3402984	-3.388948326	0.70835159	0.5070979
D:10-D:6	1.4206232	-0.628026777	3.46927314	0.4189651
P:10-D:6	-0.4511171	-2.499767082	1.59753283	0.9998651
D:12-D:6	1.6168248	-0.431825186	3.66547473	0.2400819
P:12-D:6	-0.7186725	-2.767322488	1.32997743	0.9871414
D:8-P:6	2.8307237	0.782073740	4.87937365	0.0015745
P:8-P:6	0.7265103	-1.322139694	2.77516022	0.9859177
D:10-P:6	3.4874318	1.438781856	5.53608177	0.0000731
P:10-P:6	1.6156915	-0.432958449	3.66434146	0.2409357
D:12-P:6	3.6836334	1.634983447	5.73228336	0.0000294
P:12-P:6	1.3481361	-0.700513855	3.39678606	0.4982522
P:8-D:8	-2.1042134	-4.152863391	-0.05556348	0.0398597
D:10-D:8	0.6567081	-1.391941841	2.70535807	0.9941452
P:10-D:8	-1.2150322	-3.263682146	0.83361777	0.6507810
D:12-D:8	0.8529097	-1.195740250	2.90155966	0.9512413
P:12-D:8	-1.4825876	-3.531237552	0.56606236	0.3560730
D:10-P:8	2.7609215	0.712271593	4.80957151	0.0021761
P:10-P:8	0.8891812	-1.159468712	2.93783120	0.9348984
D:12-P:8	2.9571231	0.908473184	5.00577310	0.0008737
P:12-P:8	0.6216258	-1.427024118	2.67027580	0.9964654
P:10-D:10	-1.8717403	-3.920390262	0.17690965	0.0995792
D:12-D:10	0.1962016	-1.852448366	2.24485155	1.0000000
P:12-D:10	-2.1392957	-4.187945668	-0.09064575	0.0344694
D:12-P:10	2.0679419	0.019291939	4.11659185	0.0462391
P:12-P:10	-0.2675554	-2.316205363	1.78109455	0.9999997
P:12-D:12	-2.3354973	-4.384147259	-0.28684734	0.0148944

Supplemental table 2.6.A: Post-hoc analysis results for *LeRBOH1* gene expression data. Post-hoc analysis results for gene expression data using the Tukey's Honest Significant Difference method. Results are displayed as a table with columns "Comparison" giving each combination of compared factors pairs (P for proximal, D for distal and 0-12 for timepoints), "Difference" giving the difference in the observed means, "Lower" giving the lower end point of the interval, "Upper" giving the upper end point and "Adjusted *p*-value" giving the *p*-value after adjustment for the multiple comparisons.

Comparison	Difference	Lower	Upper	Adjusted p-value
P:0-D:0	-0.71018990	-2.6872379	1.2668581	0.9843326
D:2-D:0	3.61450195	1.6374539	5.5915500	0.0000221
P:2-D:0	3.22537474	1.2483267	5.2024228	0.0001441
D:4-D:0	4.27921159	2.3021636	6.2562596	0.0000010
P:4-D:0	4.00710225	2.0300542	5.9841503	0.0000035
D:6-D:0	3.95073866	1.9736906	5.9277867	0.0000045
P:6-D:0	3.57387285	1.5968248	5.5509209	0.0000269
D:8-D:0	4.13618765	2.1591396	6.1132357	0.0000019
P:8-D:0	3.67247562	1.6954276	5.6495237	0.0000168
D:10-D:0	4.29948500	2.3224370	6.2765330	0.0000009
P:10-D:0	3.79872078	1.8216727	5.7757688	0.0000092
D:12-D:0	3.54287668	1.5658286	5.5199247	0.0000312
P:12-D:0	3.07722696	1.1001789	5.0542750	0.0002956
D:2-P:0	4.32469185	2.3476438	6.3017399	0.0000008
P:2-P:0	3.93556464	1.9585166	5.9126127	0.0000049
D:4-P:0	4.98940149	3.0123535	6.9664495	0.0000000
P:4-P:0	4.71729215	2.7402441	6.6943402	0.0000001
D:6-P:0	4.66092856	2.6838805	6.6379766	0.0000002
P:6-P:0	4.28406274	2.3070147	6.2611108	0.0000010
D:8-P:0	4.84637755	2.8693295	6.8234256	0.0000001
P:8-P:0	4.38266552	2.4056175	6.3597136	0.0000006
D:10-P:0	5.00967490	3.0326269	6.9867229	0.0000000
P:10-P:0	4.50891068	2.5318626	6.4859587	0.0000004
D:12-P:0	4.25306657	2.2760185	6.2301146	0.0000011
P:12-P:0	3.78741686	1.8103688	5.7644649	0.0000097
P:2-D:2	-0.38912721	-2.3661753	1.5879208	0.9999612
D:4-D:2	0.66470964	-1.3123384	2.6417577	0.9910637
P:4-D:2	0.39260030	-1.5844477	2.3696483	0.9999571
D:6-D:2	0.33623671	-1.6408113	2.3132847	0.9999928
P:6-D:2	-0.04062911	-2.0176771	1.9364189	1.0000000
D:8-D:2	0.52168570	-1.4553623	2.4987337	0.9990945
P:8-D:2	0.05797367	-1.9190744	2.0350217	1.0000000
D:10-D:2	0.68498305	-1.2920650	2.6620311	0.9884259
P:10-D:2	0.18421882	-1.7928292	2.1612669	1.0000000
D:12-D:2	-0.07162528	-2.0486733	1.9054228	1.0000000
P:12-D:2	-0.53727499	-2.5143230	1.4397730	0.9987830
D:4-P:2	1.05383685	-0.9232112	3.0308849	0.7827467
P:4-P:2	0.78172751	-1.1953205	2.7587756	0.9665759
D:6-P:2	0.72536392	-1.2516841	2.7024120	0.9813770
P:6-P:2	0.34849810	-1.6285499	2.3255461	0.9999890

D:8-P:2	0.91081291	-1.0662351	2.8878609	0.9040220
P:8-P:2	0.44710088	-1.5299472	2.4241489	0.9998199
D:10-P:2	1.07411026	-0.9029378	3.0511583	0.7617119
P:10-P:2	0.57334604	-1.4037020	2.5503941	0.9976999
D:12-P:2	0.31750193	-1.6595461	2.2945500	0.9999963
P:12-P:2	-0.14814778	-2.1251958	1.8289003	1.0000000
P:4-D:4	-0.27210934	-2.2491574	1.7049387	0.9999994
D:6-D:4	-0.32847293	-2.3055210	1.6485751	0.9999945
P:6-D:4	-0.70533875	-2.6823868	1.2717093	0.9851965
D:8-D:4	-0.14302394	-2.1200720	1.8340241	1.0000000
P:8-D:4	-0.60673597	-2.5837840	1.3703121	0.9960715
D:10-D:4	0.02027341	-1.9567746	1.9973215	1.0000000
P:10-D:4	-0.48049081	-2.4575389	1.4965572	0.9996123
D:12-D:4	-0.73633492	-2.7133830	1.2407131	0.9789874
P:12-D:4	-1.20198463	-3.1790327	0.7750634	0.6158983
D:6-P:4	-0.05636359	-2.0334116	1.9206844	1.0000000
P:6-P:4	-0.43322941	-2.4102774	1.5438186	0.9998721
D:8-P:4	0.12908540	-1.8479626	2.1061334	1.0000000
P:8-P:4	-0.33462663	-2.3116747	1.6424214	0.9999932
D:10-P:4	0.29238275	-1.6846653	2.2694308	0.9999986
P:10-P:4	-0.20838148	-2.1854295	1.7686666	1.0000000
D:12-P:4	-0.46422558	-2.4412736	1.5128225	0.9997306
P:12-P:4	-0.92987529	-2.9069233	1.0471727	0.8909548
P:6-D:6	-0.37686581	-2.3539139	1.6001822	0.9999730
D:8-D:6	0.18544899	-1.7915990	2.1624970	1.0000000
P:8-D:6	-0.27826303	-2.2553111	1.6987850	0.9999992
D:10-D:6	0.34874634	-1.6283017	2.3257944	0.9999889
P:10-D:6	-0.15201788	-2.1290659	1.8250302	1.0000000
D:12-D:6	-0.40786198	-2.3849100	1.5691861	0.9999342
P:12-D:6	-0.87351170	-2.8505597	1.1035363	0.9266657
D:8-P:6	0.56231480	-1.4147332	2.5393628	0.9980940
P:8-P:6	0.09860278	-1.8784453	2.0756508	1.0000000
D:10-P:6	0.72561216	-1.2514359	2.7026602	0.9813253
P:10-P:6	0.22484793	-1.7522001	2.2018960	0.9999999
D:12-P:6	-0.03099617	-2.0080442	1.9460519	1.0000000
P:12-P:6	-0.49664588	-2.4736939	1.4804022	0.9994527
P:8-D:8	-0.46371203	-2.4407601	1.5133360	0.9997337
D:10-D:8	0.16329735	-1.8137507	2.1403454	1.0000000
P:10-D:8	-0.33746687	-2.3145149	1.6395812	0.9999924
D:12-D:8	-0.59331097	-2.5703590	1.3837371	0.9968136
P:12-D:8	-1.05896069	-3.0360087	0.9180873	0.7775049
D:10-P:8	0.62700938	-1.3500387	2.6040574	0.9946860
P:10-P:8	0.12624515	-1.8508029	2.1032932	1.0000000
D:12-P:8	-0.12959895	-2.1066470	1.8474491	1.0000000
P:12-P:8	-0.59524866	-2.5722967	1.3817994	0.9967143
P:10-D:10	-0.50076423	-2.4778123	1.4762838	0.9994039
D:12-D:10	-0.75660833	-2.7336564	1.2204397	0.9739698
P:12-D:10	-1.22225804	-3.1993061	0.7547900	0.5916924
D:12-P:10	-0.25584410	-2.2328921	1.7212039	0.9999997
P:12-P:10	-0.72149382	-2.6985419	1.2555542	0.9821683
P:12-D:12	-0.46564971	-2.4426978	1.5113983	0.9997217

Supplemental table 2.7.A: Post-hoc analysis results for *LeTAPG1* gene expression data. Post-hoc analysis results for gene expression data using the Tukey's Honest Significant Difference method. Results are displayed as a table with columns "Comparison" giving each combination of compared factors pairs (P for proximal, D for distal and 0-12 for timepoints), "Difference" giving the difference in the observed means, "Lower" giving the lower end point of the interval, "Upper" giving the upper end point and "Adjusted *p*-value" giving the *p*-value after adjustment for the multiple comparisons.

Comparison	Difference	Lower	Upper	Adjusted p-value
P:0-D:0	-0.71018990	-2.6872379	1.2668581	0.9843326
D:2-D:0	3.61450195	1.6374539	5.5915500	0.0000221
P:2-D:0	3.22537474	1.2483267	5.2024228	0.0001441
D:4-D:0	4.27921159	2.3021636	6.2562596	0.0000010
P:4-D:0	4.00710225	2.0300542	5.9841503	0.0000035
D:6-D:0	3.95073866	1.9736906	5.9277867	0.0000045
P:6-D:0	3.57387285	1.5968248	5.5509209	0.0000269
D:8-D:0	4.13618765	2.1591396	6.1132357	0.0000019
P:8-D:0	3.67247562	1.6954276	5.6495237	0.0000168
D:10-D:0	4.29948500	2.3224370	6.2765330	0.0000009
P:10-D:0	3.79872078	1.8216727	5.7757688	0.0000092
D:12-D:0	3.54287668	1.5658286	5.5199247	0.0000312
P:12-D:0	3.07722696	1.1001789	5.0542750	0.0002956
D:2-P:0	4.32469185	2.3476438	6.3017399	0.0000008
P:2-P:0	3.93556464	1.9585166	5.9126127	0.0000049
D:4-P:0	4.98940149	3.0123535	6.9664495	0.0000000
P:4-P:0	4.71729215	2.7402441	6.6943402	0.0000001
D:6-P:0	4.66092856	2.6838805	6.6379766	0.0000002
P:6-P:0	4.28406274	2.3070147	6.2611108	0.0000010
D:8-P:0	4.84637755	2.8693295	6.8234256	0.0000001
P:8-P:0	4.38266552	2.4056175	6.3597136	0.0000006
D:10-P:0	5.00967490	3.0326269	6.9867229	0.0000000
P:10-P:0	4.50891068	2.5318626	6.4859587	0.0000004
D:12-P:0	4.25306657	2.2760185	6.2301146	0.0000011
P:12-P:0	3.78741686	1.8103688	5.7644649	0.0000097
P:2-D:2	-0.38912721	-2.3661753	1.5879208	0.9999612
D:4-D:2	0.66470964	-1.3123384	2.6417577	0.9910637
P:4-D:2	0.39260030	-1.5844477	2.3696483	0.9999571
D:6-D:2	0.33623671	-1.6408113	2.3132847	0.9999928
P:6-D:2	-0.04062911	-2.0176771	1.9364189	1.0000000
D:8-D:2	0.52168570	-1.4553623	2.4987337	0.9990945
P:8-D:2	0.05797367	-1.9190744	2.0350217	1.0000000
D:10-D:2	0.68498305	-1.2920650	2.6620311	0.9884259
P:10-D:2	0.18421882	-1.7928292	2.1612669	1.0000000
D:12-D:2	-0.07162528	-2.0486733	1.9054228	1.0000000
P:12-D:2	-0.53727499	-2.5143230	1.4397730	0.9987830
D:4-P:2	1.05383685	-0.9232112	3.0308849	0.7827467
P:4-P:2	0.78172751	-1.1953205	2.7587756	0.9665759
D:6-P:2	0.72536392	-1.2516841	2.7024120	0.9813770
P:6-P:2	0.34849810	-1.6285499	2.3255461	0.9999890
D:8-P:2	0.91081291	-1.0662351	2.8878609	0.9040220
P:8-P:2	0.44710088	-1.5299472	2.4241489	0.9998199

D:10-P:2	1.07411026	-0.9029378	3.0511583	0.7617119
P:10-P:2	0.57334604	-1.4037020	2.5503941	0.9976999
D:12-P:2	0.31750193	-1.6595461	2.2945500	0.9999963
P:12-P:2	-0.14814778	-2.1251958	1.8289003	1.0000000
P:4-D:4	-0.27210934	-2.2491574	1.7049387	0.9999994
D:6-D:4	-0.32847293	-2.3055210	1.6485751	0.9999945
P:6-D:4	-0.70533875	-2.6823868	1.2717093	0.9851965
D:8-D:4	-0.14302394	-2.1200720	1.8340241	1.0000000
P:8-D:4	-0.60673597	-2.5837840	1.3703121	0.9960715
D:10-D:4	0.02027341	-1.9567746	1.9973215	1.0000000
P:10-D:4	-0.48049081	-2.4575389	1.4965572	0.9996123
D:12-D:4	-0.73633492	-2.7133830	1.2407131	0.9789874
P:12-D:4	-1.20198463	-3.1790327	0.7750634	0.6158983
D:6-P:4	-0.05636359	-2.0334116	1.9206844	1.0000000
P:6-P:4	-0.43322941	-2.4102774	1.5438186	0.9998721
D:8-P:4	0.12908540	-1.8479626	2.1061334	1.0000000
P:8-P:4	-0.33462663	-2.3116747	1.6424214	0.9999932
D:10-P:4	0.29238275	-1.6846653	2.2694308	0.9999986
P:10-P:4	-0.20838148	-2.1854295	1.7686666	1.0000000
D:12-P:4	-0.46422558	-2.4412736	1.5128225	0.9997306
P:12-P:4	-0.92987529	-2.9069233	1.0471727	0.8909548
P:6-D:6	-0.37686581	-2.3539139	1.6001822	0.9999730
D:8-D:6	0.18544899	-1.7915990	2.1624970	1.0000000
P:8-D:6	-0.27826303	-2.2553111	1.6987850	0.9999992
D:10-D:6	0.34874634	-1.6283017	2.3257944	0.9999889
P:10-D:6	-0.15201788	-2.1290659	1.8250302	1.0000000
D:12-D:6	-0.40786198	-2.3849100	1.5691861	0.9999342
P:12-D:6	-0.87351170	-2.8505597	1.1035363	0.9266657
D:8-P:6	0.56231480	-1.4147332	2.5393628	0.9980940
P:8-P:6	0.09860278	-1.8784453	2.0756508	1.0000000
D:10-P:6	0.72561216	-1.2514359	2.7026602	0.9813253
P:10-P:6	0.22484793	-1.7522001	2.2018960	0.9999999
D:12-P:6	-0.03099617	-2.0080442	1.9460519	1.0000000
P:12-P:6	-0.49664588	-2.4736939	1.4804022	0.9994527
P:8-D:8	-0.46371203	-2.4407601	1.5133360	0.9997337
D:10-D:8	0.16329735	-1.8137507	2.1403454	1.0000000
P:10-D:8	-0.33746687	-2.3145149	1.6395812	0.9999924
D:12-D:8	-0.59331097	-2.5703590	1.3837371	0.9968136
P:12-D:8	-1.05896069	-3.0360087	0.9180873	0.7775049
D:10-P:8	0.62700938	-1.3500387	2.6040574	0.9946860
P:10-P:8	0.12624515	-1.8508029	2.1032932	1.0000000
D:12-P:8	-0.12959895	-2.1066470	1.8474491	1.0000000
P:12-P:8	-0.59524866	-2.5722967	1.3817994	0.9967143
P:10-D:10	-0.50076423	-2.4778123	1.4762838	0.9994039
D:12-D:10	-0.75660833	-2.7336564	1.2204397	0.9739698
P:12-D:10	-1.22225804	-3.1993061	0.7547900	0.5916924
D:12-P:10	-0.25584410	-2.2328921	1.7212039	0.9999997
P:12-P:10	-0.72149382	-2.6985419	1.2555542	0.9821683
P:12-D:12	-0.46564971	-2.4426978	1.5113983	0.9997217

Supplemental table 2.8.A: Post-hoc analysis results for *LeTAPG4* gene expression data. Post-hoc analysis results for gene expression data using the Tukey's Honest Significant Difference method. Results are displayed as a table with columns "Comparison" giving each combination of compared factors pairs (P for proximal, D for distal and 0-12 for timepoints), "Difference" giving the difference in the observed means, "Lower" giving the lower end point of the interval, "Upper" giving the upper end point and "Adjusted *p*-value" giving the *p*-value after adjustment for the multiple comparisons.

Comparison	Difference	Lower	Upper	Adjusted *p*-value
P:0-D:0	-0.71018990	-2.6872379	1.2668581	0.9843326
D:2-D:0	3.61450195	1.6374539	5.5915500	0.0000221
P:2-D:0	3.22537474	1.2483267	5.2024228	0.0001441
D:4-D:0	4.27921159	2.3021636	6.2562596	0.0000010
P:4-D:0	4.00710225	2.0300542	5.9841503	0.0000035
D:6-D:0	3.95073866	1.9736906	5.9277867	0.0000045
P:6-D:0	3.57387285	1.5968248	5.5509209	0.0000269
D:8-D:0	4.13618765	2.1591396	6.1132357	0.0000019
P:8-D:0	3.67247562	1.6954276	5.6495237	0.0000168
D:10-D:0	4.29948500	2.3224370	6.2765330	0.0000009
P:10-D:0	3.79872078	1.8216727	5.7757688	0.0000092
D:12-D:0	3.54287668	1.5658286	5.5199247	0.0000312
P:12-D:0	3.07722696	1.1001789	5.0542750	0.0002956
D:2-P:0	4.32469185	2.3476438	6.3017399	0.0000008
P:2-P:0	3.93556464	1.9585166	5.9126127	0.0000049
D:4-P:0	4.98940149	3.0123535	6.9664495	0.0000000
P:4-P:0	4.71729215	2.7402441	6.6943402	0.0000001
D:6-P:0	4.66092856	2.6838805	6.6379766	0.0000002
P:6-P:0	4.28406274	2.3070147	6.2611108	0.0000010
D:8-P:0	4.84637755	2.8693295	6.8234256	0.0000001
P:8-P:0	4.38266552	2.4056175	6.3597136	0.0000006
D:10-P:0	5.00967490	3.0326269	6.9867229	0.0000000
P:10-P:0	4.50891068	2.5318626	6.4859587	0.0000004
D:12-P:0	4.25306657	2.2760185	6.2301146	0.0000011
P:12-P:0	3.78741686	1.8103688	5.7644649	0.0000097
P:2-D:2	-0.38912721	-2.3661753	1.5879208	0.9999612
D:4-D:2	0.66470964	-1.3123384	2.6417577	0.9910637
P:4-D:2	0.39260030	-1.5844477	2.3696483	0.9999571
D:6-D:2	0.33623671	-1.6408113	2.3132847	0.9999928
P:6-D:2	-0.04062911	-2.0176771	1.9364189	1.0000000
D:8-D:2	0.52168570	-1.4553623	2.4987337	0.9990945
P:8-D:2	0.05797367	-1.9190744	2.0350217	1.0000000
D:10-D:2	0.68498305	-1.2920650	2.6620311	0.9884259
P:10-D:2	0.18421882	-1.7928292	2.1612669	1.0000000
D:12-D:2	-0.07162528	-2.0486733	1.9054228	1.0000000
P:12-D:2	-0.53727499	-2.5143230	1.4397730	0.9987830
D:4-P:2	1.05383685	-0.9232112	3.0308849	0.7827467
P:4-P:2	0.78172751	-1.1953205	2.7587756	0.9665759
D:6-P:2	0.72536392	-1.2516841	2.7024120	0.9813770

P:8-P:0	4.38266552	2.4056175	6.3597136	0.0000006
D:10-P:0	5.00967490	3.0326269	6.9867229	0.0000000
P:10-P:0	4.50891068	2.5318626	6.4859587	0.0000004
D:12-P:0	4.25306657	2.2760185	6.2301146	0.0000011
P:12-P:0	3.78741686	1.8103688	5.7644649	0.0000097
P:2-D:2	-0.38912721	-2.3661753	1.5879208	0.9999612
D:4-D:2	0.66470964	-1.3123384	2.6417577	0.9910637
P:4-D:2	0.39260030	-1.5844477	2.3696483	0.9999571
D:6-D:2	0.33623671	-1.6408113	2.3132847	0.9999928
P:6-D:2	-0.04062911	-2.0176771	1.9364189	1.0000000
D:8-D:2	0.52168570	-1.4553623	2.4987337	0.9990945
P:8-D:2	0.05797367	-1.9190744	2.0350217	1.0000000
D:10-D:2	0.68498305	-1.2920650	2.6620311	0.9884259
P:10-D:2	0.18421882	-1.7928292	2.1612669	1.0000000
D:12-D:2	-0.07162528	-2.0486733	1.9054228	1.0000000
P:12-D:2	-0.53727499	-2.5143230	1.4397730	0.9987830
D:4-P:2	1.05383685	-0.9232112	3.0308849	0.7827467
P:4-P:2	0.78172751	-1.1953205	2.7587756	0.9665759
D:6-P:2	0.72536392	-1.2516841	2.7024120	0.9813770
P:6-P:2	0.34849810	-1.6285499	2.3255461	0.9999890
D:8-P:2	0.91081291	-1.0662351	2.8878609	0.9040220
P:8-P:2	0.44710088	-1.5299472	2.4241489	0.9998199
D:10-P:2	1.07411026	-0.9029378	3.0511583	0.7617119
P:10-P:2	0.57334604	-1.4037020	2.5503941	0.9976999
D:12-P:2	0.31750193	-1.6595461	2.2945500	0.9999963
P:12-P:2	-0.14814778	-2.1251958	1.8289003	1.0000000
P:4-D:4	-0.27210934	-2.2491574	1.7049387	0.9999994
D:6-D:4	-0.32847293	-2.3055210	1.6485751	0.9999945
P:6-D:4	-0.70533875	-2.6823868	1.2717093	0.9851965
D:8-D:4	-0.14302394	-2.1200720	1.8340241	1.0000000
P:8-D:4	-0.60673597	-2.5837840	1.3703121	0.9960715
D:10-D:4	0.02027341	-1.9567746	1.9973215	1.0000000
P:10-D:4	-0.48049081	-2.4575389	1.4965572	0.9996123
D:12-D:4	-0.73633492	-2.7133830	1.2407131	0.9789874
P:12-D:4	-1.20198463	-3.1790327	0.7750634	0.6158983
D:6-P:4	-0.05636359	-2.0334116	1.9206844	1.0000000
P:6-P:4	-0.43322941	-2.4102774	1.5438186	0.9998721
D:8-P:4	0.12908540	-1.8479626	2.1061334	1.0000000
P:8-P:4	-0.33462663	-2.3116747	1.6424214	0.9999932
D:10-P:4	0.29238275	-1.6846653	2.2694308	0.9999986
P:10-P:4	-0.20838148	-2.1854295	1.7686666	1.0000000
D:12-P:4	-0.46422558	-2.4412736	1.5128225	0.9997306
P:12-P:4	-0.92987529	-2.9069233	1.0471727	0.8909548
P:6-D:6	-0.37686581	-2.3539139	1.6001822	0.9999730
D:8-D:6	0.18544899	-1.7915990	2.1624970	1.0000000
P:8-D:6	-0.27826303	-2.2553111	1.6987850	0.9999992

D:10-D:6	0.34874634	-1.6283017	2.3257944	0.9999889
P:10-D:6	-0.15201788	-2.1290659	1.8250302	1.0000000
D:12-D:6	-0.40786198	-2.3849100	1.5691861	0.9999342
P:12-D:6	-0.87351170	-2.8505597	1.1035363	0.9266657
D:8-P:6	0.56231480	-1.4147332	2.5393628	0.9980940
P:8-P:6	0.09860278	-1.8784453	2.0756508	1.0000000
D:10-P:6	0.72561216	-1.2514359	2.7026602	0.9813253
P:10-P:6	0.22484793	-1.7522001	2.2018960	0.9999999
D:12-P:6	-0.03099617	-2.0080442	1.9460519	1.0000000
P:12-P:6	-0.49664588	-2.4736939	1.4804022	0.9994527
P:8-D:8	-0.46371203	-2.4407601	1.5133360	0.9997337
D:10-D:8	0.16329735	-1.8137507	2.1403454	1.0000000
P:10-D:8	-0.33746687	-2.3145149	1.6395812	0.9999924
D:12-D:8	-0.59331097	-2.5703590	1.3837371	0.9968136
P:12-D:8	-1.05896069	-3.0360087	0.9180873	0.7775049
D:10-P:8	0.62700938	-1.3500387	2.6040574	0.9946860
P:10-P:8	0.12624515	-1.8508029	2.1032932	1.0000000
D:12-P:8	-0.12959895	-2.1066470	1.8474491	1.0000000
P:12-P:8	-0.59524866	-2.5722967	1.3817994	0.9967143
P:10-D:10	-0.50076423	-2.4778123	1.4762838	0.9994039
D:12-D:10	-0.75660833	-2.7336564	1.2204397	0.9739698
P:12-D:10	-1.22225804	-3.1993061	0.7547900	0.5916924
D:12-P:10	-0.25584410	-2.2328921	1.7212039	0.9999997
P:12-P:10	-0.72149382	-2.6985419	1.2555542	0.9821683
P:12-D:12	-0.46564971	-2.4426978	1.5113983	0.9997217

Supplementary Table S2.B: Two-way ANOVA of gene expression values in hand cut samples treated with 1-MCP. Two-way ANOVA was used to examine the e˙ects of time after induction (0 an 8 hours after induction), abscission zone side (proximal vs. distal) and presence or absence of 1-MCP treatment on the expression of the selected genes. Significance levels for the e˙ects of factors and their interaction on gene expression are given. Statistical significance was set at $p < 0.05$.

	p-value						
Gene	Factor 1 (induction)	Factor 2 (inhibitor)	Factor 3 (zone)	Interaction (1 and 2)	Interaction (1 and 3)	Interaction (2 and 3)	Interaction (1, 2 and 3)
ACO1	<0.001	<0.001	0.003	0.493	0.611	0.242	0.853
ACO4	<0.001	0.177	0.014	<0.001	0.143	0.759	0.356
EIL2	0.51473	<0.001	0.183	0.031	0.302	0.599	0.082
LX	<0.001	0.003	0.152	0.019	0.076	0.934	0.669
TBN1	<0.001	0.898	0.186	0.308	0.005	0.903	0.188
RBOH1	<0.001	0.087	0.084	0.101	0.984	0.235	0.479
TAPG1	<0.001	<0.001	0.167	0.001	0.658	0.188	0.137
TAPG4	<0.001	0.013	0.478	0.947	0.304	0.425	0.911

Supplemental Table 2.1.B: Post-hoc analysis results for *LeACO1* gene expression data in hand cut samples treated with 1-MCP.

Post-hoc analysis results for gene expression data using the Tukey's Honest Significant Difference method. Results are displayed as a table with columns "Comparison" giving each combination of compared factors pairs (P for prox-imal, D for distal, 0 or 8 for hours post induction and +MCP or -MCP for presence or absence of 1-MCP treatment), "Difference" giving the difference in the observed means, "Lower" giving the lower end point of the interval, "Upper" giving the upper end point and "Adjusted p-value" giving the p-value after ad-justment for the multiple comparisons. Statistical significance was set at $p < 0.05$.

Comparison	Difference	Lower	Upper	p adjusted
8:-MCP:D-0:-MCP:D	2.9368066	1.68169646	4.1919167	0.0000105
0:+MCP:D-0:-MCP:D	-1.8317242	-3.08683426	-0.5766141	0.0023095
8:+MCP:D-0:-MCP:D	1.2910408	0.03593066	2.5461509	0.0414945
0:-MCP:P-0:-MCP:D	-0.7201630	-1.97527312	0.5349471	0.5185132
8:-MCP:P-0:-MCP:D	1.9598354	0.70472530	3.2149455	0.0011743
0:+MCP:P-0:-MCP:D	-2.1798919	-3.43500199	-0.9247818	0.0003774
8:+MCP:P-0:-MCP:D	0.8226972	-0.43241286	2.0778073	0.3650729
0:+MCP:D-8:-MCP:D	-4.7685307	-6.02364082	-3.5134206	0.0000000
8:+MCP:D-8:-MCP:D	-1.6457658	-2.90087590	-0.3906557	0.0062488
0:-MCP:P-8:-MCP:D	-3.6569696	-4.91207968	-2.4018595	0.0000006
8:-MCP:P-8:-MCP:D	-0.9769712	-2.23208126	0.2781389	0.1934357
0:+MCP:P-8:-MCP:D	-5.1166985	-6.37180855	-3.8615884	0.0000000
8:+MCP:P-8:-MCP:D	-2.1141093	-3.36921943	-0.8589992	0.0005278
8:+MCP:D-0:+MCP:D	3.1227649	1.86765482	4.3778750	0.0000047
0:-MCP:P-0:+MCP:D	1.1115611	-0.14354896	2.3666712	0.1030012
8:-MCP:P-0:+MCP:D	3.7915596	2.53644946	5.0466697	0.0000003
0:+MCP:P-0:+MCP:D	-0.3481677	-1.60327783	0.9069424	0.9740137
8:+MCP:P-0:+MCP:D	2.6544214	1.39931129	3.9095315	0.0000376
0:-MCP:P-8:+MCP:D	-2.0112038	-3.26631387	-0.7560937	0.0008980
8:-MCP:P-8:+MCP:D	0.6687946	-0.58631545	1.9239047	0.6021174
0:+MCP:P-8:+MCP:D	-3.4709326	-4.72604274	-2.2158226	0.0000011
8:+MCP:P-8:+MCP:D	-0.4683435	-1.72345362	0.7867666	0.8891874
8:-MCP:P-0:-MCP:P	2.6799984	1.42488832	3.9351085	0.0000334
0:+MCP:P-0:-MCP:P	-1.4597289	-2.71483897	-0.2046188	0.0169856
8:+MCP:P-0:-MCP:P	1.5428603	0.28775016	2.7979703	0.0108712
0:+MCP:P-8:-MCP:P	-4.1397273	-5.39483739	-2.8846172	0.0000001
8:+MCP:P-8:-MCP:P	-1.1371382	-2.39224826	0.1179719	0.0908419
8:+MCP:P-0:+MCP:P	3.0025891	1.74747903	4.2576992	0.0000079

Supplemental Table 2.2.B: Post-hoc analysis results for *LeACO4* gene expression data in hand cut samples treated with 1-MCP.

Post-hoc analysis results for gene expression data using the Tukey's Honest Significant Difference method. Results are displayed as a table with columns "Comparison" giving each combination of compared factors pairs (P for prox-imal, D for distal, 0 or 8 for hours post induction and +MCP or -MCP for presence or absence of 1-MCP treatment), "Difference" giving the difference in the observed means, "Lower" giving the lower end point of the interval, "Upper" giving the upper end point and "Adjusted p-value" giving the p-value after ad-justment for the multiple comparisons. Statistical significance was set at $p < 0.05$.

Comparison	Difference	Lower	Upper	p adjusted
8:-MCP:D-0:-MCP:D	-2.4382241	-3.73762148	-1.13882669	0.0001573
0:+MCP:D-0:-MCP:D	-0.6958436	-1.99524096	0.60355383	0.5966508
8:+MCP:D-0:-MCP:D	-1.0953185	-2.39471592	0.20407887	0.1332630
0:-MCP:P-0:-MCP:D	-0.3481960	-1.64759336	0.95120143	0.9784478
8:-MCP:P-0:-MCP:D	-3.0077862	-4.30718358	-1.70838879	0.0000121
0:+MCP:P-0:-MCP:D	-0.8046181	-2.10401549	0.49477930	0.4300427
8:+MCP:P-0:-MCP:D	-2.1385649	-3.43796233	-0.83916754	0.0006771
0:+MCP:D-8:-MCP:D	1.7423805	0.44298312	3.04177791	0.0051155
8:+MCP:D-8:-MCP:D	1.3429056	0.04350816	2.64230295	0.0401959
0:-MCP:P-8:-MCP:D	2.0900281	0.79063072	3.38942551	0.0008633
8:-MCP:P-8:-MCP:D	-0.5695621	-1.86895950	0.72983529	0.7880312
0:+MCP:P-8:-MCP:D	1.6336060	0.33420859	2.93300338	0.0090019
8:+MCP:P-8:-MCP:D	0.2996591	-0.99973825	1.59905655	0.9907566
8:+MCP:D-0:+MCP:D	-0.3994750	-1.69887235	0.89992244	0.9556014
0:-MCP:P-0:+MCP:D	0.3476476	-0.95174979	1.64704500	0.9786324
8:-MCP:P-0:+MCP:D	-2.3119426	-3.61134001	-1.01254522	0.0002884
0:+MCP:P-0:+MCP:D	-0.1087745	-1.40817193	1.19062287	0.9999868
8:+MCP:P-0:+MCP:D	-1.4427214	-2.74211876	-0.14332397	0.0241790
0:-MCP:P-8:+MCP:D	0.7471226	-0.55227484	2.04651996	0.5161180
8:-MCP:P-8:+MCP:D	-1.9124677	-3.21186506	-0.61307026	0.0021263
0:+MCP:P-8:+MCP:D	0.2907004	-1.00869697	1.59009782	0.9922587
8:+MCP:P-8:+MCP:D	-1.0432464	-2.34264380	0.25615099	0.1683952
8:-MCP:P-0:-MCP:P	-2.6595902	-3.95898762	-1.36019282	0.0000561
0:+MCP:P-0:-MCP:P	-0.4564221	-1.75581953	0.84297526	0.9154814
8:+MCP:P-0:-MCP:P	-1.7903690	-3.08976636	-0.49097157	0.0039891
0:+MCP:P-8:-MCP:P	2.2031681	0.90377069	3.50256548	0.0004914
8:+MCP:P-8:-MCP:P	0.8692213	-0.43017614	2.16861865	0.3424914
8:+MCP:P-0:+MCP:P	-1.3339468	-2.63334423	-0.03454944	0.0420516

Supplemental Table 2.3.B: Post-hoc analysis results for *LeEIL2* gene expression data in hand cut samples treated with 1-MCP.

Post-hoc analysis results for gene expression data using the Tukey's Honest Significant Difference method. Results are displayed as a table with columns "Comparison" giving each combination of compared factors pairs (P for prox-imal, D for distal, 0 or 8 for hours post induction and +MCP or -MCP for presence or absence of 1-MCP treatment), "Difference" giving the difference in the observed means, "Lower" giving the lower end point of the interval, "Upper" giving the upper end point and "Adjusted p-value" giving the p-value after ad-justment for the multiple comparisons. Statistical significance was set at $p < 0.05$.

Comparison	Difference	Lower	Upper	p adjusted
8:-MCP:D-0:-MCP:D	-0.587637142	-1.27044400	0.09516972	0.1198958
0:+MCP:D-0:-MCP:D	0.116459006	-0.56634786	0.79926587	0.9985251
8:+MCP:D-0:-MCP:D	0.362456997	-0.32034986	1.04526386	0.6062837
0:-MCP:P-0:-MCP:D	-0.097814036	-0.78062090	0.58499283	0.9995185
8:-MCP:P-0:-MCP:D	-0.109505630	-0.79231249	0.57330123	0.9990026
0:+MCP:P-0:-MCP:D	0.278665160	-0.40414170	0.96147202	0.8388496
8:+MCP:P-0:-MCP:D	0.369189727	-0.31361713	1.05199659	0.5859869
0:+MCP:D-8:-MCP:D	0.704096148	0.02128929	1.38690301	0.0408072
8:+MCP:D-8:-MCP:D	0.950094139	0.26728728	1.63290100	0.0036404
0:-MCP:P-8:-MCP:D	0.489823106	-0.19298376	1.17262997	0.2688739
8:-MCP:P-8:-MCP:D	0.478131512	-0.20467535	1.16093837	0.2935144
0:+MCP:P-8:-MCP:D	0.866302301	0.18349544	1.54910916	0.0083272
8:+MCP:P-8:-MCP:D	0.956826868	0.27402001	1.63963373	0.0034072
8:+MCP:D-0:+MCP:D	0.245997991	-0.43680887	0.92880485	0.9051553
0:-MCP:P-0:+MCP:D	-0.214273042	-0.89707990	0.46853382	0.9507765
8:-MCP:P-0:+MCP:D	-0.225964636	-0.90877150	0.45684223	0.9361408
0:+MCP:P-0:+MCP:D	0.162206154	-0.52060071	0.84501302	0.9890279
8:+MCP:P-0:+MCP:D	0.252730721	-0.43007614	0.93553758	0.8930541
0:-MCP:P-8:+MCP:D	-0.460271033	-1.14307789	0.22253583	0.3340986
8:-MCP:P-8:+MCP:D	-0.471962628	-1.15476949	0.21084423	0.3071331
0:+MCP:P-8:+MCP:D	-0.083791838	-0.76659870	0.59901502	0.9998259
8:+MCP:P-8:+MCP:D	0.006732729	-0.67607413	0.68953959	1.0000000
8:-MCP:P-0:-MCP:P	-0.011691594	-0.69449846	0.67111527	1.0000000
0:+MCP:P-0:-MCP:P	0.376479195	-0.30632767	1.05928606	0.5640659
8:+MCP:P-0:-MCP:P	0.467003762	-0.21580310	1.14981062	0.3183870
0:+MCP:P-8:-MCP:P	0.388170790	-0.29463607	1.07097765	0.5291883
8:+MCP:P-8:-MCP:P	0.478695357	-0.20411150	1.16150222	0.2922908
8:+MCP:P-0:+MCP:P	0.090524567	-0.59228229	0.77333143	0.9997099

Supplemental Table 2.3.B: Post-hoc analysis results for *LeLX* gene expression data in hand cut samples treated with 1-MCP.

Post-hoc analysis results for gene expression data using the Tukey's Honest Significant Difference method. Results are displayed as a table with columns "Comparison" giving each combination of compared factors pairs (P for prox-imal, D for distal, 0 or 8 for hours post induction and +MCP or -MCP for presence or absence of 1-MCP treatment), "Difference" giving the difference in the observed means, "Lower" giving the lower end point of the interval, "Upper" giving the upper end point and "Adjusted p-value" giving the p-value after ad-justment for the multiple comparisons. Statistical significance was set at $p < 0.05$.

Comparison	Difference	Lower	Upper	p adjusted
8:-MCP:D-0:-MCP:D	5.87603273	3.859288	7.89277768	0.0000006
0:+MCP:D-0:-MCP:D	-0.37123418	-2.387979	1.64551077	0.9976218
8:+MCP:D-0:-MCP:D	4.25069779	2.233953	6.26744274	0.0000392
0:-MCP:P-0:-MCP:D	0.01218608	-2.004559	2.02893103	1.0000000
8:-MCP:P-0:-MCP:D	5.03798489	3.021240	7.05472984	0.0000045
0:+MCP:P-0:-MCP:D	-0.15419045	-2.170935	1.86255450	0.9999929
8:+MCP:P-0:-MCP:D	3.10938291	1.092638	5.12612786	0.0013370
0:+MCP:D-8:-MCP:D	-6.24726690	-8.264012	-4.23052195	0.0000002
8:+MCP:D-8:-MCP:D	-1.62533494	-3.642080	0.39141001	0.1654711
0:-MCP:P-8:-MCP:D	-5.86384665	-7.880592	-3.84710170	0.0000006
8:-MCP:P-8:-MCP:D	-0.83804784	-2.854793	1.17869712	0.8269340
0:+MCP:P-8:-MCP:D	-6.03022318	-8.046968	-4.01347823	0.0000004
8:+MCP:P-8:-MCP:D	-2.76664982	-4.783395	-0.74990487	0.0041536
8:+MCP:D-0:+MCP:D	4.62193197	2.605187	6.63867692	0.0000137
0:-MCP:P-0:+MCP:D	0.38342025	-1.633325	2.40016521	0.9970958
8:-MCP:P-0:+MCP:D	5.40921907	3.392474	7.42596402	0.0000017
0:+MCP:P-0:+MCP:D	0.21704373	-1.799701	2.23378868	0.9999275
8:+MCP:P-0:+MCP:D	3.48061708	1.463872	5.49736204	0.0004047
0:-MCP:P-8:+MCP:D	-4.23851171	-6.255257	-2.22176676	0.0000406
8:-MCP:P-8:+MCP:D	0.78728710	-1.229458	2.80403205	0.8656228
0:+MCP:P-8:+MCP:D	-4.40488824	-6.421633	-2.38814329	0.0000252
8:+MCP:P-8:+MCP:D	-1.14131488	-3.158060	0.87543007	0.5343999
8:-MCP:P-0:-MCP:P	5.02579881	3.009054	7.04254377	0.0000046
0:+MCP:P-0:-MCP:P	-0.16637653	-2.183121	1.85036842	0.9999880
8:+MCP:P-0:-MCP:P	3.09719683	1.080452	5.11394178	0.0013914
0:+MCP:P-8:-MCP:P	-5.19217534	-7.208920	-3.17543039	0.0000030
8:+MCP:P-8:-MCP:P	-1.92860198	-3.945347	0.08814297	0.0662147
8:+MCP:P-0:+MCP:P	3.26357336	1.246828	5.28031831	0.0008100

Supplemental Table 2.3.B: Post-hoc analysis results for *LeTBN1* gene expression data in hand cut samples treated with 1-MCP.

Post-hoc analysis results for gene expression data using the Tukey's Honest Significant Difference method. Results are displayed as a table with columns "Comparison" giving each combination of compared factors pairs (P for prox-imal, D for distal, 0 or 8 for hours post induction and +MCP or -MCP for presence or absence of 1-MCP treatment), "Difference" giving the difference in the observed means, "Lower" giving the lower end point of the interval, "Upper" giving the upper end point and "Adjusted p-value" giving the p-value after ad-justment for the multiple comparisons. Statistical significance was set at $p < 0.05$.

Comparison	Difference	Lower	Upper	p adjusted
8:-MCP:D-0:-MCP:D	90.776667	16.454436	165.098897	0.0114642
0:MCP:D-0:-MCP:D	0.240000	-74.082230	74.562230	1.0000000
8:MCP:D-0:-MCP:D	68.516667	-5.805564	142.838897	0.0822836
0:-MCP:P-0:-MCP:D	15.523333	-58.798897	89.845564	0.9948528
8:-MCP:P-0:-MCP:D	10.036667	-64.285564	84.358897	0.9996726
0:MCP:P-0:-MCP:D	1.433333	-72.888897	75.755564	1.0000000
8:MCP:P-0:-MCP:D	22.436667	-51.885564	96.758897	0.9595264
0:MCP:D-8:-MCP:D	-90.536667	-164.858897	-16.214436	0.0117168
8:MCP:D-8:-MCP:D	-22.260000	-96.582230	52.062230	0.9611331
0:-MCP:P-8:-MCP:D	-75.253333	-149.575564	-0.931103	0.0460921
8:-MCP:P-8:-MCP:D	-80.740000	-155.062230	-6.417770	0.0283574
0:MCP:P-8:-MCP:D	-89.343333	-163.665564	-15.021103	0.0130568
8:MCP:P-8:-MCP:D	-68.340000	-142.662230	5.982230	0.0835161
8:MCP:D-0:MCP:D	68.276667	-6.045564	142.598897	0.0839621
0:-MCP:P-0:MCP:D	15.283333	-59.038897	89.605564	0.9953136
8:-MCP:P-0:MCP:D	9.796667	-64.525564	84.118897	0.9997207
0:MCP:P-0:MCP:D	1.193333	-73.128897	75.515564	1.0000000
8:MCP:P-0:MCP:D	22.196667	-52.125564	96.518897	0.9616982
0:-MCP:P-8:MCP:D	-52.993333	-127.315564	21.328897	0.2749820
8:-MCP:P-8:MCP:D	-58.480000	-132.802230	15.842230	0.1844684
0:MCP:P-8:MCP:D	-67.083333	-141.405564	7.238897	0.0927793
8:MCP:P-8:MCP:D	-46.080000	-120.402230	28.242230	0.4285879
8:-MCP:P-0:-MCP:P	-5.486667	-79.808897	68.835564	0.9999944
0:MCP:P-0:-MCP:P	-14.090000	-88.412230	60.232230	0.9971462
8:MCP:P-0:-MCP:P	6.913333	-67.408897	81.235564	0.9999729
0:MCP:P-8:-MCP:P	-8.603333	-82.925564	65.718897	0.9998820
8:MCP:P-8:-MCP:P	12.400000	-61.922230	86.722230	0.9987172
8:MCP:P-0:MCP:P	21.003333	-53.318897	95.325564	0.9713083

Supplemental Table 2.3.B: Post-hoc analysis results for *LeRBOH1* gene expression data in hand cut samples treated with 1-MCP.

Post-hoc analysis results for gene expression data using the Tukey's Honest Significant Difference method. Results are displayed as a table with columns "Comparison" giving each combination of compared factors pairs (P for prox-imal, D for distal, 0 or 8 for hours post induction and +MCP or -MCP for presence or absence of 1-MCP treatment), "Difference" giving the difference in the observed means, "Lower" giving the lower end point of the interval, "Upper" giving the upper end point and "Adjusted p-value" giving the p-value after ad-justment for the multiple comparisons. Statistical significance was set at $p < 0.05$.

Comparison	Difference	Lower	Upper	p adjusted
8:-MCP:D-0:-MCP:D	2.36717507	-0.10346490	4.8378150	0.0654482
0:+MCP:D-0:-MCP:D	0.66857415	-1.80206583	3.1392141	0.9772667
8:+MCP:D-0:-MCP:D	1.27622696	-1.19441302	3.7468669	0.6356388
0:-MCP:P-0:-MCP:D	1.34946700	-1.12117297	3.8201070	0.5746676
8:-MCP:P-0:-MCP:D	3.21314749	0.74250752	5.6837875	0.0067178
0:+MCP:P-0:-MCP:D	0.62054276	-1.85009721	3.0911827	0.9849415
8:+MCP:P-0:-MCP:D	1.76030861	-0.71033137	4.2309486	0.2757342
0:+MCP:D-8:-MCP:D	-1.69860092	-4.16924090	0.7720390	0.3128249
8:+MCP:D-8:-MCP:D	-1.09094811	-3.56158809	1.3796919	0.7822399
0:-MCP:P-8:-MCP:D	-1.01770807	-3.48834804	1.4529319	0.8327977
8:-MCP:P-8:-MCP:D	0.84597242	-1.62466755	3.3166124	0.9249189
0:+MCP:P-8:-MCP:D	-1.74663231	-4.21727228	0.7240077	0.2836735
8:+MCP:P-8:-MCP:D	-0.60686646	-3.07750644	1.8637735	0.9867215
8:+MCP:D-0:+MCP:D	0.60765281	-1.86298716	3.0782928	0.9866236
0:-MCP:P-0:+MCP:D	0.68089285	-1.78974712	3.1515328	0.9749095
8:-MCP:P-0:+MCP:D	2.54457335	0.07393337	5.0152133	0.0411437
0:+MCP:P-0:+MCP:D	-0.04803139	-2.51867136	2.4226086	1.0000000
8:+MCP:P-0:+MCP:D	1.09173446	-1.37890551	3.5623744	0.7816672
0:-MCP:P-8:+MCP:D	0.07324004	-2.39739993	2.5438800	1.0000000
8:-MCP:P-8:+MCP:D	1.93692054	-0.53371944	4.4075605	0.1874743
0:+MCP:P-8:+MCP:D	-0.65568420	-3.12632417	1.8149558	0.9795568
8:+MCP:P-8:+MCP:D	0.48408165	-1.98655832	2.9547216	0.9965058
8:-MCP:P-0:-MCP:P	1.86368049	-0.60695948	4.3343205	0.2209062
0:+MCP:P-0:-MCP:P	-0.72892424	-3.19956421	1.7417157	0.9640357
8:+MCP:P-0:-MCP:P	0.41084161	-2.05979837	2.8814816	0.9987438
0:+MCP:P-8:-MCP:P	-2.59260473	-5.06324471	-0.1219648	0.0362142
8:+MCP:P-8:-MCP:P	-1.45283889	-3.92347886	1.0178011	0.4900794
8:+MCP:P-0:+MCP:P	1.13976585	-1.33087413	3.6104058	0.7456644

Supplemental Table 2.3.B: Post-hoc analysis results for *LeTAPG1* gene expression data in hand cut samples treated with 1-MCP.

Post-hoc analysis results for gene expression data using the Tukey's Honest Significant Difference method. Results are displayed as a table with columns ”Comparison” giving each combination of compared factors pairs (P for prox-imal, D for distal, 0 or 8 for hours post induction and +MCP or -MCP for presence or absence of 1-MCP treatment), ”Difference” giving the difference in the observed means, ”Lower” giving the lower end point of the interval, ”Upper” giving the upper end point and ”Adjusted p-value” giving the p-value after ad-justment for the multiple comparisons. Statistical significance was set at $p < 0.05$.

Comparison	Difference	Lower	Upper	p adjusted
8:-MCP:D-0:-MCP:D	8.96419179	4.459626	13.468758	0.0000787
0:+MCP:D-0:-MCP:D	0.70912700	-3.795439	5.213693	0.9991144
8:+MCP:D-0:-MCP:D	2.52695390	-1.977612	7.031520	0.5444435
0:-MCP:P-0:-MCP:D	3.14958009	-1.354986	7.654146	0.2950708
8:-MCP:P-0:-MCP:D	9.48717810	4.982612	13.991744	0.0000396
0:+MCP:P-0:-MCP:D	0.03122849	-4.473338	4.535795	1.0000000
8:+MCP:P-0:-MCP:D	3.30146000	-1.203106	7.806026	0.2478324
0:+MCP:D-8:-MCP:D	-8.25506479	-12.759631	-3.750499	0.0002064
8:+MCP:D-8:-MCP:D	-6.43723789	-10.941804	-1.932672	0.0028291
0:-MCP:P-8:-MCP:D	-5.81461169	-10.319178	-1.310046	0.0071730
8:-MCP:P-8:-MCP:D	0.52298631	-3.981580	5.027552	0.9998796
0:+MCP:P-8:-MCP:D	-8.93296329	-13.437529	-4.428397	0.0000821
8:+MCP:P-8:-MCP:D	-5.66273178	-10.167298	-1.158166	0.0090076
8:+MCP:D-0:+MCP:D	1.81782690	-2.686739	6.322393	0.8459708
0:-MCP:P-0:+MCP:D	2.44045309	-2.064113	6.945019	0.5837679
8:-MCP:P-0:+MCP:D	8.77805110	4.273485	13.282617	0.0001011
0:+MCP:P-0:+MCP:D	-0.67789851	-5.182465	3.826668	0.9993372
8:+MCP:P-0:+MCP:D	2.59233300	-1.912233	7.096899	0.5150860
0:-MCP:P-8:+MCP:D	0.62262619	-3.881940	5.127192	0.9996187
8:-MCP:P-8:+MCP:D	6.96022420	2.455658	11.464790	0.0013077
0:+MCP:P-8:+MCP:D	-2.49572541	-7.000292	2.008841	0.5585928
8:+MCP:P-8:+MCP:D	0.77450610	-3.730060	5.279072	0.9984482
8:-MCP:P-0:-MCP:P	6.33759801	1.833032	10.842164	0.0032812
0:+MCP:P-0:-MCP:P	-3.11835160	-7.622918	1.386215	0.3055209
8:+MCP:P-0:-MCP:P	0.15187991	-4.352686	4.656446	1.0000000
0:+MCP:P-8:-MCP:P	-9.45594961	-13.960516	-4.951383	0.0000412
8:+MCP:P-8:-MCP:P	-6.18571810	-10.690284	-1.681152	0.0041154
8:+MCP:P-0:+MCP:P	3.27023151	-1.234335	7.774798	0.2570587

Supplemental Table 2.3.B: Post-hoc analysis results for *LeTAPG4* gene expression data in hand cut samples treated with 1-MCP.

Post-hoc analysis results for gene expression data using the Tukey's Honest Significant Difference method. Results are displayed as a table with columns "Comparison" giving each combination of compared factors pairs (P for prox-imal, D for distal, 0 or 8 for hours post induction and +MCP or -MCP for presence or absence of 1-MCP treatment), "Difference" giving the difference in the observed means, "Lower" giving the lower end point of the interval, "Upper" giving the upper end point and "Adjusted p-value" giving the p-value after ad-justment for the multiple comparisons. Statistical significance was set at $p < 0.05$.

Comparison	Difference	Lower	Upper	p adjusted
8:-MCP:D-0:-MCP:D	6.1376162	0.6344371	11.6407954	0.0233878
0:+MCP:D-0:-MCP:D	-3.0099547	-8.5131339	2.4932244	0.5731340
8:+MCP:D-0:-MCP:D	3.4144723	-2.0887069	8.9176514	0.4277456
0:-MCP:P-0:-MCP:D	-1.0077482	-6.5109274	4.4954309	0.9976975
8:-MCP:P-0:-MCP:D	6.9986782	1.4954990	12.5018573	0.0081593
0:+MCP:P-0:-MCP:D	-2.5360560	-8.0392351	2.9671232	0.7465937
8:+MCP:P-0:-MCP:D	5.3979540	-0.1052251	10.9011332	0.0565721
0:+MCP:D-8:-MCP:D	-9.1475709	-14.6507501	-3.6443918	0.0006089
8:+MCP:D-8:-MCP:D	-2.7231440	-8.2263231	2.7800352	0.6798700
0:-MCP:P-8:-MCP:D	-7.1453645	-12.6485436	-1.6421853	0.0068150
8:-MCP:P-8:-MCP:D	0.8610619	-4.6421172	6.3642411	0.9991484
0:+MCP:P-8:-MCP:D	-8.6736722	-14.1768513	-3.1704930	0.0010667
8:+MCP:P-8:-MCP:D	-0.7396622	-6.2428414	4.7635169	0.9996826
8:+MCP:D-0:+MCP:D	6.4244270	0.9212478	11.9276061	0.0164935
0:-MCP:P-0:+MCP:D	2.0022065	-3.5009727	7.5053856	0.9008924
8:-MCP:P-0:+MCP:D	10.0086329	4.5054537	15.5118120	0.0002251
0:+MCP:P-0:+MCP:D	0.4738988	-5.0292804	5.9770779	0.9999840
8:+MCP:P-0:+MCP:D	8.4079087	2.9047296	13.9110879	0.0014661
0:-MCP:P-8:+MCP:D	-4.4222205	-9.9253997	1.0809586	0.1677144
8:-MCP:P-8:+MCP:D	3.5842059	-1.9189732	9.0873851	0.3722630
0:+MCP:P-8:+MCP:D	-5.9505282	-11.4537074	-0.4473491	0.0293262
8:+MCP:P-8:+MCP:D	1.9834817	-3.5196974	7.4866609	0.9049774
8:-MCP:P-0:-MCP:P	8.0064264	2.5032473	13.5096056	0.0023805
0:+MCP:P-0:-MCP:P	-1.5283077	-7.0314868	3.9748714	0.9738559
8:+MCP:P-0:-MCP:P	6.4057023	0.9025231	11.9088814	0.0168751
0:+MCP:P-8:-MCP:P	-9.5347341	-15.0379133	-4.0315550	0.0003877
8:+MCP:P-8:-MCP:P	-1.6007242	-7.1039033	3.9024550	0.9665985
8:+MCP:P-0:+MCP:P	7.9340100	2.4308308	13.4371891	0.0025993
