# Supplementary material for: 1-Aminocyclopropane-1-Carboxylate Oxidase Induction in Tomato Flower Pedicel Phloem and Abscission Related Processes Are Differentially Sensitive to Ethylene
Source: Front Plant Sci. 2017 Mar 31;8:464. doi: 10.3389/fpls.2017.00464 (PMC5374216; doi:10.3389/fpls.2017.00464)
Supplement: Supplementary file 9 [file Table3.PDF]

1-aminocyclopropane-1-carboxylate oxidase induction in tomato flower pedicel phloem and abscission related processes are differentially sensitive to ethylene

Marko Chersicola, Aleš Kladnik, Magda Tušek Žnidarič, Tanja Mrak, Kristina Gruden, Marina Dermastia.

Correspondence: marina.dermastia@nib.si

Supplementary Table S3.1. The gene expression data from hand cut timeseries samples of tomato abscission zone. Gene expression values are represented as relative copy numbers of each gene, normalized to reference gene COX.

Time after induction (hours)	Replicate	Zone	ACO1	ACO4	EIL2	LX	TBN1	RBOH1	TAPG1	TAPG4
0	1	P	8.41	1281.17	120.73	2.32	7.82	1.76	0.03	0.6
0	2	P	22.95	1226.22	125.8	6.68	8.52	4.9	0.31	1.04
0	3	P	15.14	945.72	80.73	4.78	10.34	1.56	0.01	0.46
0	1	D	18.83	1563.7	126.97	4.49	12.15	3.12	0.01	0.28
0	2	D	36.32	1562.59	90.43	3.71	9.66	3.4	0.03	2.32
0	3	D	18.71	1154.5	78.06	5.3	12.76	4.24	0.01	0.03
2	1	P	108.63	543.9	99.95	12.51	6.07	4.18	0.05	349.95
2	2	P	309.54	804.258	105.57	5.67	9.62	11.68	0.01	45.76
2	3	P	311.32	560.14	98.73	6.65	7.75	7.21	0.03	252.12
2	1	D	210.75	1016.17	120.03	24.27	21.69	7.64	0.18	574.73
2	2	D	358.38	1093.04	93.86	8.08	8.12	26.32	0.14	13.79
2	3	D	311.3	953.82	91.62	20.08	16.37	11.62	0.6	178.03
4	1	P	514.8	384.26	92.94	61.51	26.55	14	17.28	1135.55
4	2	P	374	122.5	78.29	42.47	10.15	2.39	0.33	133.66
4	3	P	276.27	103.89	76.14	152.03	119.49	13.39	24.05	1092.45
4	1	D	495.74	254.36	71.83	102.7	145.18	60.91	58.53	2855.48
4	2	D	455.14	188.54	88.22	108.18	103.71	9.25	15.11	1386.46
4	3	D	415.13	162.53	80.91	252.52	235.02	4.25	3.8	56.65
6	1	P	202.53	160.97	137.74	39.67	95.34	2.72	0.52	237.99
6	2	P	259.42	178.09	90.69	51.86	73.42	8.66	24.34	879.56
6	3	P	411.25	246.45	67.24	27.14	55.49	32.25	80.06	1726.04
6	1	D	200.34	285.84	164.15	60.46	247.15	5.2	1.2	291.98
6	2	D	435.15	350.99	110.09	159.49	365.23	10.12	8.9	815.27
6	3	D	542.67	291.2	86.76	119.36	316.44	25.04	69.09	969.24
8	1	P	226	66.3	65.72	292.11	180.88	150.56	689.25	2973.62
8	2	P	449.88	90.5	55.53	223.29	108.57	86.66	471.32	1924.73
8	3	P	260.88	106.1	68.67	150.84	89.6	103.61	349.64	2575.5
8	1	D	260.35	92.29	62.75	538.35	619.81	133.56	344.11	1444.28
8	2	D	647.78	114.98	59.35	675.07	354.19	97.78	434.96	1449.58
8	3	D	412.51	176.92	49.69	338.57	637.1	91.84	205.35	1416.98
10	1	P	407.82	154.75	81.25	460.27	201.88	149.51	567.42	2162.32
10	2	P	545.05	49.17	80.61	620.84	232.88	156.55	3075.93	3488.44
10	3	P	155.15	88.35	94.84	593.3	237.79	105.93	580.76	1724.55
10	1	D	483.41	267.38	70.38	1088.01	760.18	143.88	459.54	1589.1
10	2	D	705.18	106.48	78.91	1678.11	818.79	118.84	1051.56	2157.52
10	3	D	286.6	188.98	88.92	1368.99	880.4	25.08	104.25	545.78
12	1	P	95.87	157.67	73.87	272.86	79.84	50.27	38	732.69
12	2	P	368.69	176.99	86.57	491.83	384.82	205.37	1948.83	3892.48
12	3	P	217.64	96.85	84.95	257.37	208.6	122.54	995.95	1955.54
12	1	D	174.04	176.9	63.19	978.61	656.93	59.83	85.11	691.42
12	2	D	320.1	304.54	99.84	1172.32	1198.93	185.07	591.23	2130.68
12	3	D	363.64	258.09	89.36	943.46	1046.27	82.27	444.53	1582.6

Supplementary Table S3.2. The gene expression data from hand cut and 1-MCP treated samples of tomato abscission zone. Gene expression values are represented as relative copy numbers of each gene, normalized to reference gene COX.

Time after induction (hours)	Treatment with 1-MCP	Replicates	Zone	ACO1	ACO4	EIL2	LX	TBN1	RBOH1	TAPG1	TAPG4
0	+	1	P	4.42	253.06	155.08	4.3	7.92	5.87	1.23	0.23
0	+	1	D	5.21	330.7	159.54	2.28	3.96	5.19	0.6	0.74
0	+	2	P	2.57	279.62	129.88	0.39	2.78	2.38	0.03	0.16
0	+	2	D	4.95	238.43	120.77	0.3	2.42	3.87	0.03	0.03
0	+	3	P	2.78	448.33	224.28	0.79	1.28	1.78	0.03	0.075
0	+	3	D	3.48	557.59	144.12	1.01	2.02	1.43	0.47	0.03
0	-	1	P	25.62	390.41	188.16	0.79	3.54	3.25	0.98	0.44
0	-	1	D	62.64	643.54	182.09	0.93	2.07	0.79	0.03	8.83
0	-	2	P	5.59	358.71	176.31	0.65	2.04	1.93	0.33	20.49
0	-	2	D	14.04	385.85	153.47	1.01	3.77	2.03	1.12	20.99
0	-	3	P	17.59	890.82	178.66	4.25	48.67	35.31	39.56	0.03
0	-	3	D	24.85	1427.97	207.85	2.24	1.84	2.41	0.03	0.03
8	+	1	P	103.45	84.85	84.24	29.17	18.22	12.5	7.5	770.32
8	+	1	D	101.48	234.93	116.3	124.38	84.29	18	4.16	356.86
8	+	2	P	51.88	129.58	100.56	25.1	33.7	10.95	1.52	323.47
8	+	2	D	104.66	350.87	88.84	75.17	66.94	7.54	1.25	23.6
8	+	3	P	48.05	52.75	172.36	32.33	23.07	5.55	1.77	240.71
8	+	3	D	98.96	160.9	189.51	77.7	62	1.31	0.38	18.55
8	-	1	P	251.41	54.09	116.95	233.66	17.07	53.7	1425.94	2544.2
8	-	1	D	586.65	86.63	59.25	640.24	169.64	12.29	587.43	714.79
8	-	2	P	190.33	29.7	133.11	181.55	10.48	18.99	1210.96	1528.94
8	-	3	P	163.34	26.61	90.56	181.71	10.24	58.21	1339.42	1877.65
8	-	2	D	549.44	75.94	77.52	360.21	70.42	14.94	999.35	926.04
8	-	3	D	454.53	35.88	73.13	412.99	39.95	25.55	820.51	833.46

Supplementary Table S3.3. The gene expression data from microdissected sam-ples of tomato abscission zone. Gene expression values are represented as rela-tive copy numbers of each gene, normalized to reference gene COX.

Time after induction (hours)	Treatment with 1-MCP	Tissue	ACO1	ACO4	EIL2	LX	TBN1	RBOH1	TAPG1	TAPG4
0	-	distal	5.85	112.74	56.89	0.69	14.38	18.85	13.82	0.08
0	-	distalAZ	4.51	225.52	103.79	0.69	27.03	23.48	17.43	0.08
0	-	proximalAZ	0.63	138.48	97.83	0.69	19.84	29.44	14.99	0.08
0	-	proximal	0.63	144.15	63.72	0.69	0.27	1.48	1.63	0.08
0	+	distal	7.02	202.59	86.67	0.69	21.51	36.03	22.94	0.08
0	+	distalAZ	5.68	231.67	122.9	0.69	28.38	29.38	18.79	0.08
0	+	proximalAZ	4.21	135.45	90.49	0.69	28.98	28.42	17.73	0.08
0	+	proximal	0.63	207.32	75.54	0.69	12.73	17.55	10.18	0.39
8	-	distal	69.04	21.72	60.83	138.07	252.41	28.09	23.38	74.02
8	-	distalAZ	6.61	1.86	56.72	215.34	59.37	154.24	757.63	737.22
8	-	proximalAZ	5.92	2.03	79.1	147.97	23.46	138.6	1406.41	1365.78
8	-	proximal	66.68	20.58	99.63	146.27	81.42	62.74	122.44	210.73
8	+	distal	43.21	201.49	74.16	171.75	63.42	33.69	6.78	3
8	+	distalAZ	38.86	133.65	106.97	73.33	125.68	35.03	11.35	201.5
8	+	proximalAZ	11.17	14.53	196.8	10.79	88.63	42.02	20.93	421.68
8	+	proximal	39.41	53.07	75.04	10.43	24.12	30.91	8.67	3.98
8	-	distal vein	21.3	79.16	78.04	118.47	486.53	48.32	48.81	166.89
8	-	distal pith	1.99	1.46	97.71	47.11	113.78	106.89	216.37	654.35
8	-	proximal vein	27.81	34.9	113.18	96.42	249.16	148.88	505.4	1605.06
8	-	proximal pith	1.99	1.46	134.9	37.03	4.56	4.42	203.36	935.99
